# Effectiveness of a blended multidisciplinary intervention for patients with moderate medically unexplained physical symptoms (PARASOL): A cluster randomized clinical trial

**DOI:** 10.1371/journal.pone.0283162

**Published:** 2023-04-06

**Authors:** Paula Elisabeth van Westrienen, Niek de Wit, Suze Toonders, Cindy Veenhof, Marloes Gerrits, Martijn Pisters

**Affiliations:** 1 Department of Health Innovations and Technology, Research Group Empowering Healthy Behaviour, Fontys University of Applied Sciences, Eindhoven, Netherlands; 2 Center for Physical Therapy Research and Innovation in Primary Care, Utrecht, Netherlands; 3 Department of Rehabilitation, Physical Therapy Science and Sport, Physical Therapy Research, UMC Utrecht Brain Center Utrecht University, Utrecht, Netherlands; 4 Department of General Practice, Julius Center for Health Sciences and Primary Care, University Medical Center Utrecht, Utrecht, Netherlands; 5 Research Group Innovation of Human Movement Care, University of Applied Sciences Utrecht, Utrecht, Netherlands; Amsterdam UMC Locatie VUmc, UNITED STATES

## Abstract

**Introduction:**

In patients with moderate Medically Unexplained Physical Symptoms (MUPS), interventions focusing on both physical and psychological aspects are recommended. A proactive, blended and integrated physical therapy and mental health nurse intervention (PARASOL) might reduce complaints, stimulate self-management and prevent chronicity.

**Objective:**

To investigate short- and long-term effectiveness of the PARASOL intervention compared to usual care on subjective symptom impact and quality of life in patients with moderate MUPS.

**Methods:**

We conducted a cluster randomized clinical trial. The 12-week intervention integrated face-to-face sessions with the physical therapist and mental health nurse and access to a web-based program consisting of graded activity, exercises and information modules. Primary outcomes were subjective symptom impact, as registered with the adequate relief question, and quality of life. Secondary outcomes were severity of (psychosocial) symptoms, overall current health, physical behaviour, illness perceptions, and self-management skills. Assessment took place at baseline, after three and twelve months.

**Results:**

Compared to usual care (n = 80), the number of patients in the PARASOL intervention (n = 80) that reported adequate short-term relief was higher (31.2% in intervention group vs. 13.7% in control group). On quality of life and secondary outcomes no significant between group differences in short- and long-term were found.

**Conclusions:**

The PARASOL intervention does improve subjective symptom impact of patients with moderate MUPS on short-term. No additional beneficial effects on the other outcomes and the long-term were found.

## Introduction

Medically unexplained physical symptoms (MUPS) are defined as physical complaints such as pain, fatigue and/or dizziness for which no pathophysiological explanation can be found after adequate medical examination [1–3]. MUPS is classified in a continuum from mild, to moderate, to chronic MUPS [1]. The majority (75%) of the patients have mild MUPS, in whom symptoms generally recover within 1–3 months [4–6]. These symptoms usually have a low impact in one or two domains and are in many patients transient. Twenty percent of the patients with MUPS have persisting symptoms after three months. Most of them have moderate MUPS [4], and experience severe unexplained symptoms in two or three domains with a higher impact in daily life, with psychological and physical distress, but without a diagnosis of a functional somatic syndrome (FSS), or a somatic symptom disorder (SSD) according to the Diagnostic and Statistical Manual of Mental Disorders, 5th edition [4,7]. Patients with moderate MUPS experience a better quality of life than patients with chronic MUPS [8]. The remaining 5% have chronic MUPS, defined by the presence of FSS, such as fibromyalgia, chronic fatigue syndrome or irritable bowel syndrome, or SSD [4,7]. Patients with chronic MUPS have more severe symptoms and experience psychological and physical dysfunction [4]. Chronic MUPS has a high impact on patients’ quality of life and daily functioning [9,10], and are associated with mental health disorders as depression and anxiety [11]. MUPS can be clarified by the biopsychosocial model, based on the theory of Engel [12]. Physical, psychological and social factors are considered to be contributors to the symptoms and experienced disability.

Many patients with MUPS have a good prognosis. Fifty to 75% of the patients with MUPS improve within one year. Yet, unfortunately approximately 10 to 30% deteriorate [13]. An unfavourable course towards chronicity is expected when patients have a multiple number of physical symptoms within different clusters, experience more severe symptoms, have poorer physical functioning, have financial problems or have a history of childhood physical abuse [13,14]. Furthermore, female gender and an older age seems to be associated with unfavourable disease course, but results are inconsistent [14,15].

Much research has been conducted on effective interventions for chronic MUPS. Neurosciences-based therapeutic education, cognitive behavioral therapy, and exercise therapy have been shown to be effective treatment modalities in patients with chronic MUPS [16–19]. Most pharmacological interventions focused on antidepressants in patients with chronic MUPS. No pharmacological interventions are known that sufficiently treat all symptoms while avoiding the risk of adverse events [20]. The guideline therefore recommend to be reluctant with pharmacotherapy in MUPS [2]. Overall, the vast majority of these studies included patients with chronic MUPS. So far little research has been conducted in patients with moderate MUPS.

For GPs, adequate management of chronic MUPS is challenging, given the unexplained background and high consultation frequency [10,21]. For society, the high health care utilization in chronic MUPS creates a financial burden [10,22]. Because of the high impact of chronic MUPS there is a need for early identification of patients with moderate MUPS and (cost-)effective interventions to prevent chronicity.

We demonstrated in earlier research that patients with moderate MUPS can be adequately identified using data of the electronic medical records of the GP [23]. Subsequently patients can be proactively approached by the GP for intervention. However, so far, no effective interventions for patients with moderate MUPS are known. Currently, Dutch multidisciplinary guidelines recommend focus on both physical and mental aspects in treatment [2]. In the Dutch gatekeeper system, patients consult their GP first. Within Dutch general practices, GPs work together with a mental health nurse. Mental health nurses work under the supervision of the GP [24]. They have received higher vocational training in nursing or psychology and deliver short-term care to patients with psychosocial problems [24]. The GP is suggested to act as case manager, in close collaboration with the mental health nurse and/or physical therapist with a special interest in MUPS. So far solid evidence for effectiveness of this integrated approach is lacking [2,25].

We developed a proactive, blended and integrated multidisciplinary intervention (PARASOL) for patients with moderate MUPS in primary care with the aim to prevent chronicity [26]. The intervention integrates face-to-face sessions with the physical therapist and mental health nurse with a web-based program of graded activity, information modules and exercises. This blended care approach provides patients 24/7 access to an online eCoachings platform, ensuring continuity of care and encouragement of self-management. Therefore, the aim of this study was to evaluate the effectiveness of the PARASOL intervention on subjective symptom impact and quality of life of patients with moderate MUPS in primary care, as compared to usual care. As our intervention can be considered a complex intervention, the Medical Research Council framework was used for the evaluation of PARASOL [27].

## Materials and methods

### Design

A prospective, multicenter cluster randomized clinical trial in primary care, reported according to the CONSORT Cluster Trial checklist.

### Setting and participants

Fifteen multidisciplinary health care centers, with in total 110.000 patients, participated. Patients were eligible if they were 18 years or older, had at least five GP’s consultations in the past 12 months, of which three or more resulted in a diagnosis suggestive of MUPS. Furthermore, patients with an established chronic MUPS diagnosis (i.e. fibromyalgia, chronic fatigue syndrome or irritable bowel syndrome) and a confirmed medical and/or psychiatric diagnosis (i.e. chronic obstructive pulmonary disease, hypertension or diabetes mellitus schizophrenia, anxiety disorder or depressive disorder) were excluded.

Eligible patients were approached using three strategies [23]. In the first strategy patients with moderate MUPS were identified in the electronic medical records of the GP using the previously reported PRESUME screening method [23]. All identified patients were proactively approached with an invitation letter of their GP explaining the study. In the second strategy participating GPs of the fifteen health care centers actively recruited patients with moderate MUPS during consultations, and–if met the PRESUME criteria for moderate MUPS–linked the patient to the research group for inclusion. In the third strategy patients were recruited through flyers in the waiting rooms in the fifteen participating health care centers by placing and study information in the centers’ newsletters. Patients who were willing to participate were encouraged to contact the researcher by phone or by mail. Subsequently, the researcher checked the diagnosis (moderate MUPS according to the PRESUME criteria), and confirmed that patients had access to internet and master the Dutch language. After receiving detailed information about the study’s aims and procedures, patients were asked to provide written informed consent.

### Intervention program

**Fig 1:**
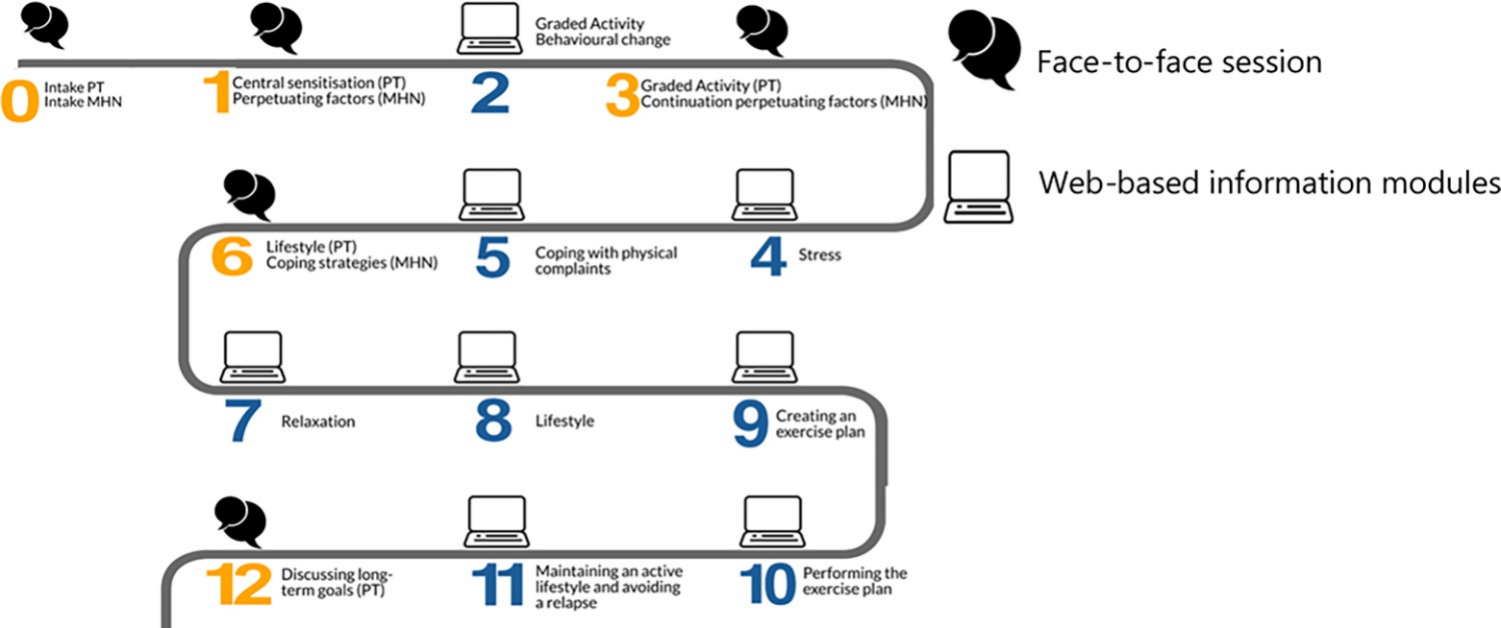
Overview of the PARASOL intervention.

The twelve week PARASOL intervention integrates five face-to-face sessions with the physical therapist, four face-to-face sessions with the mental health nurse and access to a web-based program focusing on 1) graded activity, 2) exercises and 3) information modules (shown in Fig 1). The components of the intervention were based on results of a literature search and focus groups with experts (GPs, psychosomatic physical therapists, mental health nurses and health care psychologists) [28]. The structure of the web-based program was based on the e-Exercise intervention for patients with hip or knee osteoarthritis [29].

The intake started with the physical therapist since participants’ perception of the symptoms usually has a somatic focus and patients with MUPS are often reluctant to focus on the psychosocial complaints [30,31]. The physical therapist focused on the somatic complaints and conducted a physical examination to get insight in the physical status (e.g. posture and movement, breathing patterns and muscle tension). Afterwards, the physical therapist created an account for the web-based program and added symptom specific exercises in the web-based program and informed the participant about the first information modules with corresponding home assignments. In the second part of the intake, the mental health nurse focused on cognitive, emotional, behavioural and social aspects of the complaints [32]. Patients’ treatment goals and treatment demand were also identified during the intake. After completing the intake the two professionals discussed the complaints, the background, the expectations and the treatment goals of the patient.

After the intake, participants had four follow-up sessions with the physical therapist and three with the mental health nurse, combined with home assignments in the web-based program. The home assignments and the themes of the information modules (videos and descriptions) were discussed during the face-to-face sessions. Participants followed an online graded activity program and received instructions for exercises at home (shown in Fig 1). Participants received automatic support during their graded activity program and home exercises of the web-based program. Weekly automatic emails informed and reminded participants about new assignments and content. The content of the intervention was described in more detail elsewhere [26].

### Usual care

Usual care was defined as routine GP care for patients with MUPS, which could be provided by the GP, physical therapist, mental health nurse and psychologist, without restrictions. The physical therapists and mental health nurses of the health care centers in the control group were blinded to the intervention, i.e. they were not aware of the content of the PARASOL intervention. After the end of the study, participants in the control group were offered to follow the PARASOL intervention.

### Outcomes

Study outcomes were assessed at baseline, three months (short-term) and twelve months (long-term). Participants were asked to fill out questionnaires and wear an activity monitor for a week. Participant characteristics such as age, gender, marital status, education level, work situation, duration of complaints, and possible comorbidities were measured at baseline. If participants did not complete the questionnaires, a first reminder was sent after one week and a second reminder or a phone call after two weeks. No financial incentives were offered to complete the measurements.

### Primary outcome measures

We used two primary outcomes to evaluate the PARASOL intervention. The first one was subjective symptom impact, as registered with the adequate relief question. This is a validated single question measurement, which is scored on a dichotomous scale (“Over the past week have you had adequate relief of your symptoms?”) [33,34]. Adequate short-term relief was defined as a participant who reported adequate relief of their symptoms for at least six of the twelve weeks between the baseline and three-month follow-up. If not, a participant was defined as a non-responder [35]. Adequate long-term relief was defined as a participant who reported adequate relief of their symptoms for at least four of the seven months between the 6- and 12-month follow-up. The second primary outcome was quality of life, as assessed with the 36-Item Short Form Health Survey (RAND-36) health survey [36,37]. The RAND-36 consists of eight subscales, which were merged into two summary component scales: “Physical Component Scale” (PCS) and Mental Component Scale” (MCS). The norm-based score for the PCS and MCS was 50, where a score below 50 meant a less favourable physical and mental health state [38,39].

### Secondary outcome measures

Symptom severity for pain and fatigue was assessed using a numeric rating scale ranging from 0 (no pain/no fatigue) to 10 (worst possible pain/fatigue) [40]. Severity of psychosocial symptoms was assessed with the Four-Dimensional Symptom Questionnaire (4DSQ) [41,42]. The questionnaire consists of four subscales, namely distress with a score range of 0–32, depression with a score range of 0–12, anxiety with a score range of 0–24 and the somatisation scale with a score range of 0–32. A higher score defines an increased probability of a disorder. Overall current health was assessed with the EuroQol visual analogue scale (EQ VAS) [43]. Scores ranged from 0 (“the worst health you can imagine”) to 100 (“the best health you can imagine”). Physical behaviour was assessed with the Activ8 activity monitor [44]. The Activ8 is an activity monitor that measures physical behaviour by measuring several activities and postures (lying, sitting, standing, walking, running and cycling). Data were converted into total sedentary time and the average amount of hours of moderate or vigorous physical activity (MVPA). Total sedentary time (average hours per day) included any waking behaviour characterized by an energy expenditure ≤1.5 metabolic equivalents, while in a sitting, reclining or lying posture. MVPA was measured, to determine if participants met the Dutch Standard for Healthy Physical Activity criteria. Participants met the Dutch Standard for Healthy Physical Activity if they had at least 150 minutes of moderate intense physical activity every week, spread over several different days [45]. Illness perceptions were assessed with the Brief Illness Perception Questionnaire [46,47]. The questionnaire consists of eight items and has a score range of 0–10. Higher scores on personal control beliefs, treatment control beliefs and coherence beliefs indicates an improvement in perception, whereas on consequences beliefs, timeline beliefs, identity beliefs, concern beliefs and emotional response beliefs a lower score indicates an improvement in perception. Self-management skills were assessed with the Health Education Impact Questionnaire. The questionnaire consists of eight subscales and were scored on a 4-point Likert scale (“totally disagree” to “totally agree”) [48]. A higher score indicates a higher level of self-management.

### Sample size

The power calculation was based on the recommendations of Campbell et al [49] for cluster randomized trials and performed for the primary outcome measure quality of life (power = 0.8; alpha = 0.05). An intraclass correlation coefficient of 0.05 was assumed and a minimum cluster size of 20. In addition, to detect a clinically relevant difference between groups, a difference of >10 points in the sum score of physical functioning of the RAND-36 questionnaire and a SD of 23.8 were used in sample size calculation [50]. With an expected dropout rate of 20%, a total of 248 participants (n = 124 per arm) were needed.

#### Randomization

We used cluster randomization on health care center level to prevent contamination effects. Of the 15 health care centers, eight were randomized to the PARASOL intervention and seven the control group. Concealment of allocation was ensured since a person outside of the research team performed the randomization. The health care centers were informed about their allocation by email. The health care professionals and patients were not blinded. The main investigators were also not blinded to group assignment.

Physical therapists and mental health nurses of the health care centers assigned to the intervention group were asked if they had a special interest or already experience in treating patients with MUPS. The physical therapists and mental health nurses signed up were instructed how to treat patients with moderate MUPS during a two-day training on the content of the PARASOL intervention. Furthermore, a booster session after six months was conducted to ensure adherence to the treatment protocol. The physical therapists and mental health nurses of the control health care centers were not trained.

### Ethics

The trial protocol and study material was approved by the Medical Ethical Committee of University Medical Center Utrecht, the Netherlands (number 16/532). The trial was registered in the Dutch trial register with number NL6581. The authors confirm that all ongoing and related trials for this intervention are registered.

Participants were informed about the design and conduct of the study and asked for informed consent. They were assigned to a unique trial code and participant information was stored separately from outcome data.

### Statistical analysis

Descriptive statistics were used to describe participants’ general characteristics. Frequencies, t tests and chi-square tests were used to explore agreement in demographics between both groups on general characteristics. The primary analyses were performed according to the intention-to-treat principle. Per-protocol analyses were performed for participants who attended all face-to-face meetings of the PARASOL intervention and for all participants in the usual care group, despite per-protocol analyses were not planned in the initial study protocol [26]. Missing values were imputed with the Multivariate Imputation by Chained Equations.

We performed univariate and multivariate analyses to determine the effectiveness of the PARASOL intervention on mean differences in the primary and secondary outcome measures on short- and long-term. In both univariate and multivariate analyses, the baseline value was included as covariate [51]. In the multivariate analyses, we controlled for recruitment strategy, marital status, age and duration of symptoms, since these variables had a more than 10% change-in-estimate of the effect. The primary outcome subjective symptom impact was analyzed by logistic regression. All other outcome measures were analyzed with a linear regression model. From these models, we estimated the mean of the outcomes for the intervention group and control group, mean differences within groups and mean differences between groups (with 95% CIs).

To determine if linear mixed model analysis with a 2-level hierarchy was necessary, heterogeneity was assessed across health care centers on quality of life as primary outcome measure by calculating the ICC. The highest ICC was found to be 0.034. Linear mixed model analyses were performed, but no variations between clusters was observed. Therefore, only univariate and multivariate intention-to-treat analyses are presented in the tables.

Per-protocol analyses consisted of multivariate analyses controlling for the same variables as the primary analyses. Additional sensitivity analyses were performed by comparing the results of the main analysis of subjective symptom impact for different cut-off points to ensure the validity of the results. On short-term, we adjusted the cut-off points by defining a responder as a participant who reported adequate relief of their symptoms for at least 40% or at least 60% of the measurements. On long term, we adjusted the cut-off points by defining a responder as a participant who reported adequate relief of their symptoms for at least 40% or at least 70% of the measurements. Analyses were carried out using SPSS Statistics 25.0 (IBM SPSS, Chicago, Illinois).

## Results

### Participant flow

After randomisation, one health care center allocated to the intervention group declined to participate due to lack of time of the health care professionals. In the remaining 14 health centers, 169 eligible patients were included between March 2017 and April 2018. Of these, 139 (82%) participants were identified through the PRESUME approach, 5 (3%) were recruited during GP’s consultation and 25 (15%) via flyers in the waiting rooms and study information in the centers’ newsletters. On average, five participants were included per health care center (range 2 to 34).

Nine eligible patients did not provide informed consent, because of lack of time (n = 1), priority for another treatment (n = 1) or other/unknown (n = 7). Of the remaining 160 participants, 80 originated from health care centers allocated to the intervention group and 80 from health care centers allocated to the control group. The inclusion stopped after the originally planned 12 months because of the financial budget restrictions of the project.

Seven physical therapists and six mental health nurses from the health care centers allocated to the PARASOL intervention, were trained in the PARASOL intervention. On average they each treated 12 participants (range 5 to 26). No adverse effects of the intervention were reported.

**Fig 2:**
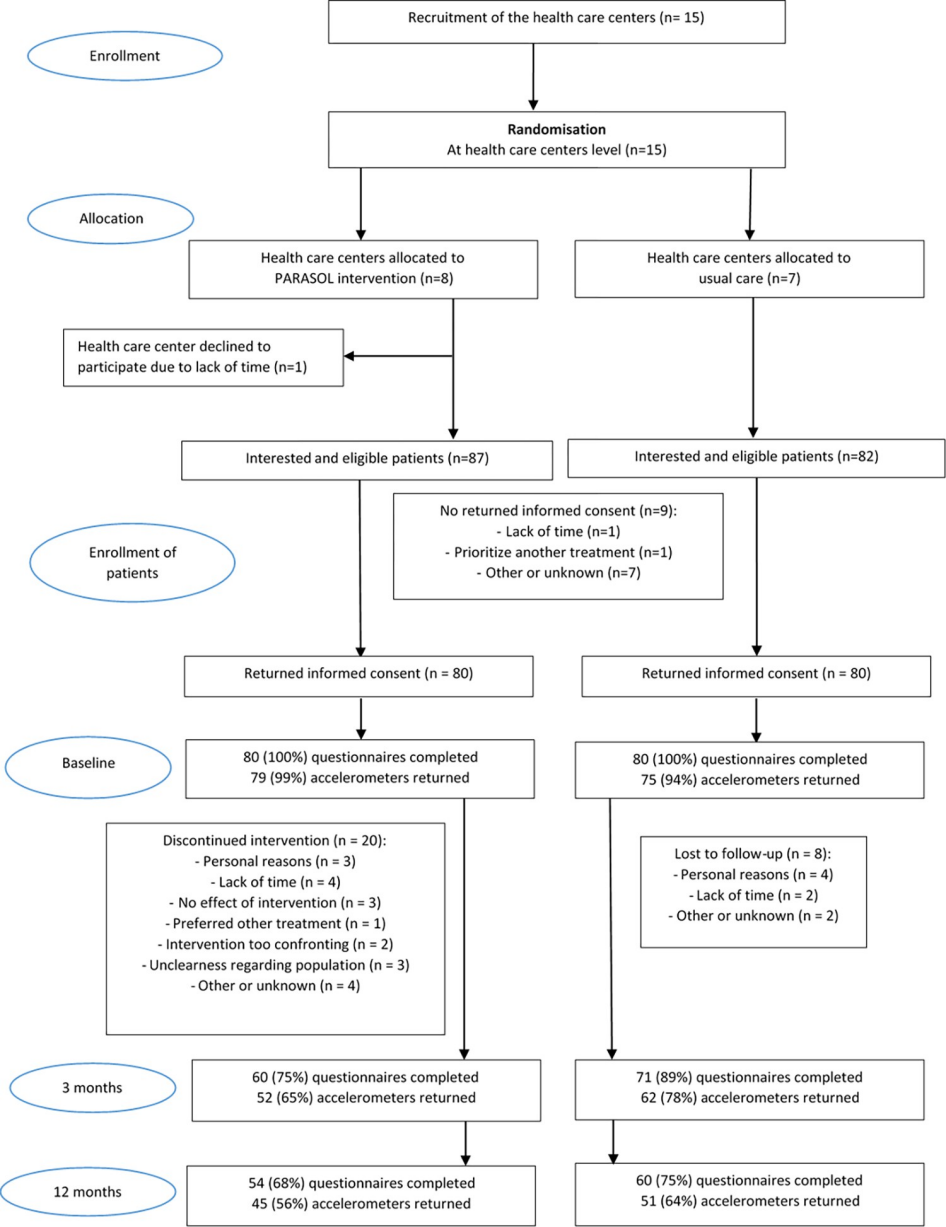
Flow chart.

The response rate for the questionnaires was 100% at baseline, 82% at three months, and 71% at twelve months (Fig 2). Eligible activity monitor data at baseline, three months and twelve months were available for 96%, 71%, and 60% of the 160 participants, respectively. Overall dropout rate in the intervention group was 33% and 25% in the control group. In the intervention group dropouts were significantly older and had a significantly shorter duration of symptoms compared to the non-drop outs. In the control group dropouts and non-dropouts did not differ. Furthermore, the two patient groups did differ significantly in recruitment strategy (Table 1).

**Table 1:** Characteristics of participants^a^.

Characteristic	Baseline		
	All participants (n = 160)	Exp (n = 80)	Con (n = 80)
Gender, female	119 (74.4)	57 (71.3)	62 (77.5)
Age *(yr)*, mean (SD)	48.4 (13.7)	47.1 (12.4)	49.7 (14.9)
Duration of symptoms			
0 mo.– 1 y.	22 (13.7)	8 (10)	14 (17.5)
≥1 y.	138 (86.3)	72 (90)	66 (82.5)
Education level			
Low	41 (25.6)	18 (22.5)	23 (28.8)
Middle	65 (40.6)	38 (47.5)	27 (33.8)
High	54 (33.8)	24 (30)	30 (37.5)
Work status			
Student	2 (1.3)	1 (1.3)	1 (1.3)
Employed	103 (64.4)	53 (66.3)	50 (62.5)
Unemployed	27 (16.9)	13 (16.3)	14 (17.5)
Retired	22 (13.8)	10 (12.5)	12 (15)
Volunteer	6 (3.8)	3 (3.8)	3 (3.8)
Marital status^b^			
Unmarried	56 (35)	22 (27.5)	34 (42.5)
Married/living with a partner	103 (64.4)	57 (71.3)	46 (57.5)
No. of comorbidities			
0	85 (53.1)	45 (56.2)	40 (50)
1	31 (19.4)	15 (18.8)	16 (20)
≥2	44 (27.5)	20 (25)	24 (30)
Recruitment strategy			
PRESUME screening	130 (81.3)	57 (71.3)	73 (91.3)
GP during consultation	5 (3.1)	5 (6.3)	0 (0)
Open recruitment	25 (15.6)	18 (22.5)	7 (8.8)

^a^Data are reported as number (percentage) of participants unless otherwise indicated.

^b^One participant included in the experimental group refused to answer here marital status.

Exp = experimental group, Con = control group.

### Short-term effectiveness

After completing the intervention, 31.2% of patients reported adequate relief, as compared to 13.7% in the control group (Table 2). This between group difference persisted after adjustment for recruitment strategy, marital status, age and duration of symptoms in multivariate analysis (Table 3).

**Table 2:** Unadjusted primary outcome measures. Mean (SD) of groups, mean (SD) difference within groups, and mean difference (95% CI) or odds ratio (95% CI) between groups.

Outcome	Groups						Difference within groups				Difference between groups	
	Week 0		3 months		12 months		3 months minus Week 0		12 months minus Week 0		3 months minus Week 0	12 months minus Week 0
	Exp (n = 80)	Con (n = 80)	Exp	Con	Exp	Con	Exp	Con	Exp	Con	Exp minus Con	Exp minus Con
Quality of Life RAND-36 *(0–100)*												
Physical Component Scale	42.7 (7.3)	42.7 (10.1)	46.5 (13.1)	43.9 (12.8)	46.5 (15.1)	43.9 (14)	MD 3.7 (1.4 to 6)	MD 1.3 (-1 to 3.5)	MD 3.7 (0.8 to 6.7)	MD 1.3 (-1.6 to 4.1)	MD 2.5 (-0.7 to 5.7)	MD 2.5 (-1.9 to 6.6)
Mental Component Scale	41.9 (12.7)	46 (11.6)	46.5 (20.1)	49 (15.2)	46.6 (17.2)	48.1 (14.7)	MD 4.6 (1.1 to 8.1)	MD 3 (-0.3 to 6.4)	MD 4.7 (1.3 to 8.1)	MD 2.1 (-1.2 to 5.3)	MD -0.3 (-5.5 to 4.8)	MD 0.4 (-4.3 to 5.1)
Impact of symptoms Adequate relief *(yes/no)*												
Responder, n(%)	..	..	25 (31.2)	11 (13.7)	33 (41.3)	25 (31.2)	..	..	..	..	OR 2.9 (1.2 to 6.9)	OR 1.6 (0.7 to 3.4)

Exp = experimental group, Con = control group.

MD = Mean Difference.

**Table 3:** Adjusted multivariate comparisons of primary and secondary outcome measures per group based on intention-to-treat analysis at 3 and 12 months.

	Difference between groups					
	3 months minus Week 0	Between- group difference (P)	Between-group effect size	12 months minus Week 0	Between- group difference (P)	Between-group effect size
	Exp minus Con			Exp minus Con		
Quality of Life RAND-36 *(0–100)* *						
Physical Component Scale	2.3 (-1.2 to 5.8)	0.19	0.17	2.9 (-1.5 to 7.3)	0.19	0.13
Mental Component Scale	0.3 (-5.1 to 5.7)	0.91	0.07	0.6 (-4.2 to 5.5)	0.79	0.11
Impact of symptoms						
Adequate relief (yes/no)†	2.8 (1.1 to 7.3)	0.04	0.18^a^	1.5 (0.7 to 3.4)	0.33	0.10^a^
Severity of symptoms NRS (0–10)*						
Pain	-0.7 (-2.3 to 0.8)	0.35	-0.10	-0.9 (-2 to 0.3)	0.13	-0.18
Fatigue	-0.7 (-2.1 to 0.7)	0.34	-0.12	-0.4 (-1.4 to 0.7)	0.51	-0.11
Severity of psychosocial symptoms 4DSQ *						
Distress *(0–32)*	-0.8 (-4.7 to 3.2)	0.71	-0.07	-0.3 (-3.7 to 3)	0.84	-0.08
Depression *(0–12)*	-0.3 (-1.4 to 0.8)	0.63	-0.09	-0.2 (-1.4 to 1)	0.78	-0.07
Anxiety *(0–24)*	-0.3 (-2 to 1.3)	0.71	-0.09	-0.2 (-1.5 to 1.1)	0.79	-0.09
Somatization *(0–32)*	-1.6 (-4.4 to 1.2)	0.27	-0.15	-1.7 (-4.5 to 1)	0.21	-0.16
Physical behaviour (h/d)*						
Sedentary behaviour	0.4 (-1 to 1.7)	0.61	0.03	0.3 (-0.7 to 1.4)	0.52	0.06
Moderate or vigorous physical activity	0.1 (-0.3 to 0.5)	0.66	0.09	-0.1 (-0.4 to 0.2)	0.37	-0.02
EQ VAS (0–100)*						
Overall current health	5.8 (-4.7 to 16.3)	0.28	0.18	3.6 (-4.2 to 11.4)	0.36	0.16
Illness perceptions IPQ-k (0–10)*						
Consequences	-0.1 (-1.7 to 1.6)	0.95	-0.05	-0.3 (-1.5 to 0.8)	0.58	-0.10
Timeline	0 (-1.9 to 1.8)	0.97	0	0.6 (-0.8 to 2)	0.42	0.08
Personal control	0.8 (-1 to 2.7)	0.37	0.10	0.7 (-0.6 to 2)	0.32	0.14
Treatment control	0.8 (-1.1 to 2.6)	0.42	0.01	0 (-1.5 to 1.5)	0.98	-0.13
Identity	0.1 (-1.2 to 1.3)	0.93	-0.03	-0.5 (-1.6 to 0.5)	0.31	-0.14
Concern	0.3 (-1.4 to 2)	0.73	-0.01	0.1 (-1.1 to 1.3)	0.83	-0.05
Coherence	0.6 (-0.9 to 2.2)	0.42	0.12	0.4 (-0.9 to 1.7)	0.53	0.15
Emotional response	0.5 (-1.2 to 2.3)	0.53	0.01	0.4 (-0.8 to 1.6)	0.51	-0.02
Self-management skills HEI-Q (1–4)*						
Health-directed activity	0.06 (-0.27 to 0.38)	0.73	0.15	-0.08 (-0.36 to 0.21)	0.61	0.14
Positive and active engagement in life	0.04 (-0.23 to 0.30)	0.78	0.06	0.10 (-0.15 to 0.36)	0.42	0.13
Self-monitoring and insight	0.18 (-0.11 to 0.47)	0.22	0.19	0.18 (-0.04 to 0.40)	0.10	0.24
Constructive attitude and approaches	-0.01 (-0.29 to 0.27)	0.93	0.04	0.06 (-0.16 to 0.28)	0.61	0.11
Skill and technique acquisition	0.18 (-0.22 to 0.58)	0.38	0.19	0.15 (-0.09 to 0.39)	0.22	0.27
Social integration and support	0.07 (-0.28 to 0.42)	0.70	0.07	-0.12 (-0.37 to 0.13)	0.35	-0.04
Emotional distress	0.01 (-0.29 to 0.31)	0.96	0.07	0.01 (-0.25 to 0.26)	0.94	0.07
Health service navigation	0.01 (-0.31 to 0.32)	0.96	0.01	-0.01 (-0.24 to 0.23)	0.95	0

Data are odds ratio (95% CI).

*Data are differences in mean (95%CI).

^a^Data are risk differences.

Exp = experimental group, Con = control group.

The quality of life of patients within the intervention group improved significantly both for PCS and MCS (Table 2). However, no between group differences in quality of life were found. Adjustment for potential confounders showed similar results (Table 3).

As for the secondary outcomes, patients within the intervention group improved significantly on overall current health, severity of psychosocial symptoms subscale distress and subscale somatization, and the illness perception items personal control, coherence, and emotional response (S1 Table). In contrast, in the usual care group, none of the outcome measures showed any significant within group differences over time. However, no significant between group differences were found on the secondary outcome measures (S1 Table). Adjustment for potential confounders showed similar results (Table 3).

Short-term results of the per-protocol analyses showed similar results on the primary outcome measures as the intention-to-treat analyses (S2 Table). Both sensitivity analyses, where different cut-off points were compared, demonstrate comparable findings on subjective symptom impact (S1 Appendix).

### Long-term effectiveness

In 12-month follow-up, the percentage of patients with adequate relief in the intervention group was 41.3%, as compared to 31.2% in the control group. Between group differences were not statistically significant after adjustment for potential confounders (Table 3).

Quality of life improved significantly on PCS and MCS in patients from the intervention group (Table 2). However, no between group differences in quality of life were found on the long-term. Adjustment for potential confounders showed similar results (Table 3).

As for the secondary outcomes, patients within the intervention group improved significantly on overall current health, severity of symptoms pain and fatigue, severity of psychosocial symptoms subscale distress, subscale anxiety and subscale somatization, and the illness perception items consequences, personal control and identity (S1 Table). Short and long-term results were similar for the secondary outcome measures. No significant between group differences were found on the secondary outcome measures (Table 3).

Long-term results of the per-protocol analyses showed similar results on the primary outcome measures as the intention-to-treat analyses. Only on the secondary outcome measures, heiQ subscale “self-monitoring and insight”, a statistically significant difference between groups was found (S2 Table). The sensitivity analyses, where different cut-off points were compared, demonstrated comparable findings on subjective symptom impact (S1 Appendix).

## Discussion

This is the first multicenter cluster randomized clinical trial of a proactive, blended and integrated intervention with a physical therapist and mental health nurse for primary care patients with moderate MUPS aiming at prevention of chronicity. The results showed more patients with short-term adequate relief after treatment with the PARASOL intervention compared to the usual care group. Unfortunately, this between group difference in favour of the PARASOL intervention did not sustain in long-term. Although quality of life improved within the PARASOL group after the intervention, this improvement did not differ from the usual care group. No additional beneficial effects of the PARASOL intervention on the secondary outcomes were found, neither in short-term nor in long-term follow-up.

Subjective symptom impact was one of the primary outcome measures because this subjective outcome adequately reflects the perception of symptoms. Better adequate short-term relief was not accompanied by significant improvements on severity of symptom scores. The explanation for this finding might be that the intervention focused on patients’ insight, perception of symptoms and modifiable prognostic risk factors. Thus the main effect of the PARASOL intervention might be diminishing the impact of symptoms on patients by improving coping strategies and perception of symptoms, without having an effect on symptom severity.

The PARASOL intervention is the first blended care intervention in patients with moderate MUPS. It is hypothesized that blended care can help to stimulate self-management. Although self-management skills improved after the intervention, this improvement did not differ from the usual care group. A possible explanation might be that patients had a lack of intrinsic motivation due to the proactive approach of the GP since the presence of motivation is an important aspect for patients’ self-management [52]. Therefore, insight in patients’ self-management skills should be assessed for personalization of the intervention, to enable stimulation of self-management in the right patients.

Although not statistically significant, a positive trend in the between group differences on quality of life in favour of the PARASOL intervention was found. Not achieving the preset sample size might be an important reason why we were not able to demonstrate the effectiveness of the PARASOL intervention. The size of the effect on quality of life at the end of treatment was similar to earlier research, but differ with a more recent primary care intervention in patients with MUPS [19,53]. Sitnikova et al. found a significant effect on the physical component of quality of life at the end of the treatment, but this effect did not sustain on long-term. This is remarkable compared to our results, since our results showed a sustained long-term improvement, although not different from the usual care group. The sustained long-term improvement might be due to the fact that the PARASOL intervention focused on adopting and maintaining a behavioural change. Taking into account the sustained long-term improvement on quality of life and the short-term effect on subjective symptom impact, despite the low power, we recommend to optimize the PARASOL intervention. An idea for optimization is adding a booster session a few weeks after the end of the intervention, to enhance long-term effectiveness and reinforce changes.

### Strengths and limitations

The strength of this study is that we opted for cluster randomization, in order to keep the effect of the intervention as pure as possible to prevent a contamination effect. The following limitations of the present study need to be taken into account. First, we only included 160 participants while the desired number of participants was 248. Since the number of interested patients was lower than expected in the first recruitment strategy, we added the second and third recruitment strategy and extended the inclusion period with another six months, but we still did not achieve our sample size needed. This may raise questions regarding the validity of our results. However, absence of evidence is not evidence of absence [54]. So it might be that the absence of a statistically significant effect is due to a small sample despite a positive trend in difference in endpoints. However, on the other hand the non-significant between group differences in quality of life on the short term and long-term differences on the primary outcomes can be considered small effect sizes. Besides the low number of participants, the inclusion of participants using the three recruitment strategies differed substantially between both groups, which may be considered as bias. Secondly, we had to deal with high drop-out rates: 18% after three months and 29% after twelve months. Percentages of missing data in our activity monitor data were even higher. The low number of participants and the high drop-out rates might be attributed to a relatively long follow-up period, the number and length of the measurements and the recruitment strategy where patients were proactively approached by the GP and therefore might be less motivated to change. Retrospectively, conducting a pilot study first might have been better in terms of recruitment success and a lower drop-out rate. Thirdly, our included patient group is very heterogeneous. Patients with moderate MUPS differ on severity of symptoms, duration of symptoms and might have varying needs. The heterogeneity might have contributed to more outliers and a wide dispersion across participants. Fourthly, the 2-day training session for the physical therapists and mental health nurses of the health care centers assigned in the intervention group was a bit short. Blended care is a relatively new way of delivering care, which requires a different way of working for health care professionals. Therefore, training of the health care professionals is a target for improvement. Future training should also have more focus on gaining insight in the added value of integrated care and how this supports patients’ self-management. Another limitation are the established baseline differences between groups on both primary and secondary outcome measures despite randomization, which might have influenced our findings [51]. Overall, the intervention group had a lower score on baseline measurements as compared to the control group. As a consequence, patients in the intervention group had a higher potential to improve. This might be attributable to the fact that only patients with more severe complaints wanted to participate, after which the symptoms generally improve during the trial, also known as regression to the mean. In addition, in both groups a proportion of the patients might have improved spontaneously [23]. A final limitation is that the psychometric properties of the adequate relief question in patients with moderate MUPS are unknown. The adequate relief question is a validated clinically relevant endpoint in patients with irritable bowel syndrome, an established chronic MUPS diagnosis. This may raise questions regarding the relevancy in patients with moderate MUPS. However, we chose the adequate relief question since this subjective outcome reflects on the perception of symptoms which is as important as actual symptom severity in patients with functional disorders [55].

## Clinical implications and future directions

The current trend in daily practice is a stepped care strategy with attention to self-management. This includes that patients are treated in accordance with their symptom severity by the right health care professional in the right place at the right time. In the Netherlands health care insurance companies require that patients follow a primary care intervention first before they can be referred to secondary care. In our opinion, the PARASOL intervention, if optimized, suits in this requirement. In case of deterioration of symptoms or unsatisfying results, patients could be referred to secondary care.

Given the adequate short-term relief and the improvements within the intervention group on short- and long-term, PARASOL has the potential to become a valuable primary care intervention. The PARASOL intervention can prevent high costs for society, including health care costs. Therefore, cost-effectiveness from a societal perspective of the PARASOL intervention compared to usual care in patients with moderate MUPS will be evaluated in another study.

## Conclusion

In conclusion, a relatively short multidisciplinary intervention in primary care, integrating face-to-face sessions with a web-based program does improve subjective symptom impact of patients with moderate MUPS on short-term. No additional beneficial effects on the other outcomes were found on the short and long-term.

## Supporting information

S1 Table Unadjusted secondary outcome measures. Mean (SD) of groups, mean (SD) difference within groups, and mean difference (95% CI) or odds ratio (95% CI) between groups.
(DOCX)

S2 Table Primary and secondary outcome measures based on per-protocol analysis at 3 and 12 months. (DOCX)

S1 File Sensitivity analyses of subjective symptom impact. (DOCX)

S2 File Approved study protocol. (PDF)

S3 File Published study protocol. This is an open access article distributed under the terms of the Creative Commons Attribution License, which permits unrestricted use, distribution, and reproduction in any medium, provided the original author and source are credited.
(PDF)

S4 File CONSORT 2010 checklist. (DOC)

S1 Appendix Sensitivity analyses of subjective symptom impact. (DOCX)
