# Supplementary material for: Effectiveness of a blended multidisciplinary intervention for patients with moderate medically unexplained physical symptoms (PARASOL): A cluster randomized clinical trial
Source: PLoS One. 2023 Apr 6;18(4):e0283162. doi: 10.1371/journal.pone.0283162 (PMC10079131; doi:10.1371/journal.pone.0283162)
Supplement: S1 Table — Mean (SD) of groups, mean (SD) difference within groups, and mean difference (95% CI) or odds ratio (95% CI) between groups. (DOCX) [file pone.0283162.s001.docx]

S1 Table. Unadjusted secondary outcome measures. Mean (SD) of groups, mean (SD) difference within groups, and mean difference (95% CI) or odds ratio (95% CI) between groups.

| Outcome | Groups | | | | | | | |  | Difference within groups | | | | |  | Difference between groups | | |
| --- | --- | --- | --- | --- | --- | --- | --- | --- | --- | --- | --- | --- | --- | --- | --- | --- | --- | --- |
|  | Week 0 | |  | 3 months | |  | 12 months | |  | 3 months minus Week 0 | |  | 12 months minus Week 0 | |  | 3 months minus Week 0 |  | 12 months minus Week 0 |
|  | Exp (n = 80) | Con (n = 80) |  | Exp (n = 80) | Con (n = 80) |  | Exp (n = 80) | Con (n = 80) |  | Exp | Con |  | Exp | Con |  | Exp minus Con |  | Exp minus Con |
| Severity of symptoms NRS *(0-10)* |  |  |  |  |  |  |  |  |  |  |  |  |  |  |  |  |  |  |
| Pain | 5.2 (2.4) | 4.8 (2.5) |  | 4.2 (5.7) | 4.5 (4.1) |  | 3.9 (3.8) | 4.5 (3.7) |  | MD -1 (-2.3 to 0.2) | MD -0.3 (-1.4 to 0.7) |  | MD -1.3 (-2.3 to -0.3) | MD -0.3 (-1.3 to 0.7) |  | MD -0.5 (-2 to 0.9) |  | MD -0.8 (-1.9 to 0.3) |
| Fatigue | 6.2 (2.6) | 5.7 (2.6) |  | 5.2 (5.3) | 5.4 (4.1) |  | 5 (2.8) | 5.1 (3.4) |  | MD -1.1 (-2.3 to 0.1) | MD -0.3 (-1.4 to 0.8) |  | MD -1.2 (-2.2 to -0.2) | MD -0.6 (-1.6 to 0.3) |  | MD -0.5 (-1.9 to 0.9) |  | MD -0.3 (-1.3 to 0.8) |
| Severity of psychosocial symptoms 4DSQ |  |  |  |  |  |  |  |  |  |  |  |  |  |  |  |  |  |  |
| Distress *(0-32)* | 13.5 (8.6) | 11.2 (8.4) |  | 10.5 (15.8) | 9.5 (11.3) |  | 10.4 (10.7) | 9.4 (10) |  | MD -3 (-5.6 to -0.4) | MD -1.7 (-4.1 to 0.7) |  | MD -3.1 (-5.4 to -0.7) | MD -1.8 (-4.1 to 0.5) |  | MD -0.5 (-4.3 to 3.4) |  | MD 0.1 (-3.1 to 3.2) |
| Depression *(0-12)* | 2 (3) | 1.3 (2.6) |  | 1.4 (4.6) | 1.1 (3.5) |  | 1.3 (4.1) | 1 (3.4) |  | MD -0.6 (-1.6 to 0.4) | MD -0.2 (-1 to 0.7) |  | MD -0.7 (-1.7 to 0.4) | MD -0.3 (-1.3 to 0.7) |  | MD -0.3 (-1.3 to 0.8) |  | MD 0 (-1.1 to 1.1) |
| Anxiety *(0-24)* | 3.2 (4.3) | 2.4 (4.5) |  | 2.2 (6.5) | 2.1 (5.3) |  | 1.8 (4.1) | 1.7 (4.3) |  | MD -1 (-2.3 to 0.4) | MD -0.3 (-1.5 to 0.8) |  | MD -1.4 (-2.7 to -0.2) | MD -0.7 (-2 to 0.6) |  | MD -0.3 (-1.9 to 1.2) |  | MD -0.1 (-1.3 to 1.1) |
| Somatization *(0-32)* | 13.4 (6.9) | 12 (6.9) |  | 10.4 (11.1) | 10.8 (8.4) |  | 9.7 (9.5) | 10.4 (9.3) |  | MD -3 (-5 to -1.1) | MD -1.2 (-3 to 0.6) |  | MD -3.7 (-5.7 to -1.6) | MD -1.6 (-3.6 to 0.4) |  | MD -1.4 (-4 to 1.2) |  | MD -1.4 (-3.9 to 1.2) |
| Physical behaviour *(h/d)* |  |  |  |  |  |  |  |  |  |  |  |  |  |  |  |  |  |  |
| Sedentary behaviour | 9 (2.4) | 9 (2.2) |  | 9.2 (5.3) | 9 (4) |  | 8.6 (3.5) | 8.3 (3.4) |  | MD 0.2 (-1.1 to 1.5) | MD 0 (-1.1 to 1.2) |  | MD -0.3 (-1.4 to 0.7) | MD -0.7 (-1.7 to 0.4) |  | MD 0.2 (-1.1 to 1.5) |  | MD 0.3 (-0.7 to 1.3) |
| Moderate or vigorous physical activity | 0.4 (0.5) | 0.6 (0.6) |  | 0.6 (1.3) | 0.6 (1) |  | 0.4 (0.8) | 0.6 (0.9) |  | MD 0.1 (-0.4 to 0.7) | MD 0 (-0.4 to 0.4) |  | MD 0 (-0.4 to 0.4) | MD 0 (-0.4 to 0.4) |  | MD 0.1 (-0.3 to 0.5) |  | MD -0.1 (-0.4 to 0.1) |
| EQ VAS *(0-100)* |  |  |  |  |  |  |  |  |  |  |  |  |  |  |  |  |  |  |
| Overall current health | 60.7 (19.3) | 67.2 (17) |  | 72.2 (38.8) | 70.5 (28.6) |  | 69.3 (26.8) | 69.6 (24) |  | MD 11.4 (5.4 to 17.5) | MD 3.2 (-2.6 to 9.1) |  | MD 8.6 (3 to 14.1) | MD 2.4 (-3.1 to 7.9) |  | MD 4.9 (-5.2 to 15) |  | MD 1.6 (-5.9 to 9.1) |
| Illness perceptions IPQ-k *(0-10)* |  |  |  |  |  |  |  |  |  |  |  |  |  |  |  |  |  |  |
| Consequences | 5.8 (2.7) | 5.2 (2.7) |  | 5.4 (5.8) | 5.2 (4.2) |  | 4.8 (3.6) | 4.7 (3.8) |  | MD -0.5 (-1.7 to 0.8) | MD -0.1 (-1.2 to 1) |  | MD -1 (-2 to -0.1) | MD -0.5 (-1.5 to 0.4) |  | MD -0.1 (-1.6 to 1.4) |  | MD -0.2 (-1.3 to 0.8) |
| Timeline | 7.4 (2.6) | 7.5 (3.3) |  | 7.3 (7.1) | 7.5 (5.3) |  | 7.3 (4.7) | 7 (4.6) |  | MD -0.1 (-1.5 to 1.3) | MD -0.1 (-1.3 to 1.2) |  | MD -0.1 (-1.2 to 1.1) | MD -0.6 (-1.7 to 0.6) |  | MD -0.1 (-1.9 to 1.7) |  | MD 0.4 (-0.9 to 1.7) |
| Personal control | 4.3 (2.3) | 4.6 (2.7) |  | 5.8 (6.4) | 5.2 (4.5) |  | 5.7 (4.1) | 5.1 (4) |  | MD 1.5 (0.1 to 2.9) | MD 0.6 (-0.6 to 1.9) |  | MD 1.4 (0.2 to 2.5) | MD 0.5 (-0.6 to 1.6) |  | MD 0.6 (-1.1 to 2.3) |  | MD 0.6 (-0.6 to 1.9) |
| Treatment control | 6 (2.2) | 4.9 (3) |  | 6.2 (6.8) | 5 (4.8) |  | 5.4 (4.6) | 5.2 (4.8) |  | MD 0.2 (-1.2 to 1.6) | MD 0.1 (-1.1 to 1.4) |  | MD -0.6 (-1.9 to 0.6) | MD 0.3 (-1 to 1.5) |  | MD 0.7 (-1 to 2.5) |  | MD 0 (-1.5 to 1.4) |
| Identity | 6.4 (2.1) | 6 (2.3) |  | 5.9 (4.5) | 5.6 (3.3) |  | 5.2 (3.6) | 5.5 (3.6) |  | MD -0.5 (-1.5 to 0.6) | MD -0.3 (-1.2 to 0.6) |  | MD -1.2 (-2.1 to -0.2) | MD -0.5 (-1.4 to 0.4) |  | MD 0 (-1.1 to 1.2) |  | MD -0.5 (-1.5 to 0.5) |
| Concern | 5.7 (2.7) | 5 (2.9) |  | 5.1 (6) | 4.5 (4.5) |  | 4.8 (4.1) | 4.4 (3.6) |  | MD -0.6 (-1.9 to 0.7) | MD -0.5 (-1.6 to 0.7) |  | MD -0.9 (-1.9 to 0.2) | MD -0.6 (-1.7 to 0.4) |  | MD 0.2 (-1.4 to 1.8) |  | MD 0.2 (-0.9 to 1.3) |
| Coherence | 5.3 (2.2) | 5.9 (3.2) |  | 6.8 (5.6) | 6.5 (4.2) |  | 6.3 (4.2) | 5.9 (4.3) |  | MD 1.5 (0.2 to 2.8) | MD 0.6 (-0.5 to 1.8) |  | MD 1 (-0.2 to 2.1) | MD 0 (-1.2 to 1.2) |  | MD 0.6 (-1 to 2.1) |  | MD 0.5 (-0.7 to 1.8) |
| Emotional response | 6.1 (2.7) | 5.2 (3.2) |  | 5.2 (6.3) | 4.2 (4.3) |  | 5.1 (3.8) | 4.3 (4) |  | MD -0.9 (-0.1 to -1.8) | MD -1 (-2.1 to 0.1) |  | MD -1 (-2 to 0.1) | MD -0.9 (-2 to 0.2) |  | MD 0.5 (-1.2 to 2.1) |  | MD 0.5 (-0.7 to 1.6) |
| Self-management skills HEI-Q *(1-4)* |  |  |  |  |  |  |  |  |  |  |  |  |  |  |  |  |  |  |
| Health-directed activity | 2.94 (0.69) | 3.27 (0.64) |  | 3.16 (1.29) | 3.29 (0.91) |  | 3.13 (0.88) | 3.27 (0.76) |  | MD 0.23 (-0.27 to 0.73) | MD 0.02 (-0.37 to 0.41) |  | MD 0.19 (-0.22 to 0.59) | MD 0 (-0.38 to 0.37) |  | MD 0.06 (-0.25 to 0.37) |  | MD -0.07 (-0.34 to 0.20) |
| Positive and active engagement in life | 2.96 (0.55) | 3.05 (0.55) |  | 3.05 (0.97) | 3.08 (0.70) |  | 3.09 (0.84) | 3.04 (0.77) |  | MD 0.09 (-0.31 to 0.50) | MD 0.02 (-0.31 to 0.35) |  | MD 0.13 (-0.26 to 0.52) | MD -0.01 (-0.36 to 0.33) |  | MD 0.04 (-0.21 to 0.28) |  | MD 0.09 (-0.15 to 0.33) |
| Self-monitoring and insight | 2.80 (0.44) | 2.93 (0.45) |  | 3.00 (1.09) | 2.90 (0.79) |  | 3.03 (0.70) | 2.91 (0.73) |  | MD 0.21 (-0.25 to 0.66) | MD -0.03 (-0.39 to 0.32) |  | MD 0.23 (-0.15 to 0.60) | MD -0.01 (-0.35 to 0.33) |  | MD 0.19 (-0.09 to 0.47) |  | MD 0.16 (-0.04 to 0.37) |
| Constructive attitude and approaches | 3.02 (0.53) | 3.13 (0.59) |  | 3.09 (1.05) | 3.16 (0.79) |  | 3.15 (0.77) | 3.15 (0.69) |  | MD 0.08 (-0.35 to 0.50) | MD 0.03 (-0.32 to 0.38) |  | MD 0.13 (-0.20 to 0.47) | MD 0.02 (-0.32 to 0.36) |  | MD 0.01 (-0.25 to 0.27) |  | MD 0.05 (-0.15 to 0.26) |
| Skill and technique acquisition | 2.51 (0.54) | 2.81 (0.58) |  | 2.90 (1.55) | 2.84 (1.06) |  | 2.86 (0.76) | 2.85 (0.76) |  | MD 0.39 (-0.18 to 0.96) | MD 0.03 (-0.42 to 0.49) |  | MD 0.35 (-0.02 to 0.71) | MD 0.04 (-0.33 to 0.41) |  | MD 0.16 (-0.21 to 0.54) |  | MD 0.21 (-0.11 to 0.35) |
| Social integration and support | 2.76 (0.67) | 2.86 (0.63) |  | 2.92 (1.31) | 2.91 (0.90) |  | 2.83 (0.87) | 2.98 (0.93) |  | MD 0.16 (-0.35 to 0.66) | MD 0.05 (-0.33 to 0.42) |  | MD 0.07 (-0.32 to 0.46) | MD 0.12 (-0.30 to 0.54) |  | MD 0.07 (-0.26 to 0.39) |  | MD -0.11 (-0.36 to 0.14) |
| Emotional distress | 2.87 (0.65) | 3.06 (0.63) |  | 3.05 (1.19) | 3.14 (0.89) |  | 3.10 (0.95) | 3.20 (0.86) |  | MD 0.17 (-0.29 to 0.64) | MD 0.08 (-0.29 to 0.45) |  | MD 0.23 (-0.17 to 0.63) | MD 0.14 (-0.25 to 0.53) |  | MD 0.04 (-0.25 to 0.32) |  | MD -0.02 (-0.26 to 0.23) |
| Health service navigation | 2.98 (0.53) | 2.99 (0.56) |  | 3.04 (1.22) | 3.03 (0.94) |  | 3.06 (0.75) | 3.07 (0.85) |  | MD 0.05 (-0.42 to 0.53) | MD 0.04 (-0.34 to 0.41) |  | MD 0.08 (-0.28 to 0.44) | MD 0.08 (-0.31 to 0.47) |  | MD 0.02 (-0.28 to 0.31) |  | MD 0 (-0.23 to 0.22) |
| Exp = experimental group, Con = control group | | |  |  |  |  |  |  |  |  |  |  |  |  |  |  |  |  |
