# Supplementary material for: Effectiveness of a blended multidisciplinary intervention for patients with moderate medically unexplained physical symptoms (PARASOL): A cluster randomized clinical trial
Source: PLoS One. 2023 Apr 6;18(4):e0283162. doi: 10.1371/journal.pone.0283162 (PMC10079131; doi:10.1371/journal.pone.0283162)
Supplement: S2 Table — (DOCX) [file pone.0283162.s002.docx]

S2 Table. Primary and secondary outcome measures based on per-protocol analysis at 3 and 12 months.

|  |  | Difference between groups | | |
| --- | --- | --- | --- | --- |
|  |  | 3 months minus Week 0 |  | 12 months minus Week 0 |
|  |  | Exp minus Con |  | Exp minus Con |
| Quality of Life RAND-36 *(0-100)* * |  |  |  |  |
| Physical Component Scale |  | 3 (0 to 6) |  | 3.1 (-1 to 7.3) |
| Mental Component Scale |  | 1 (-3.6 to 5.5) |  | 1.4 (-3.4 to 6.3) |
| Impact of symptoms |  |  |  |  |
| Adequate relief (yes/no)† |  | 2.8 (1 to 8) |  | 1.3 (0.5 to 3.2) |
| Severity of symptoms NRS *(0-10)** |  |  |  |  |
| Pain |  | -1 (-2.1 to 0.2) |  | -1 (-2.1 to 0.2) |
| Fatigue |  | -1 (-2.2 to 0.1) |  | -0.4 (-1.5 to 0.7) |
| Severity of psychosocial symptoms 4DSQ* |  |  |  |  |
| Distress *(0-32)* |  | -1.1 (-4.4 to 2.1) |  | -0.2 (-3.3 to 2.9) |
| Depression *(0-12)* |  | -0.3 (-1.3 to 0.6) |  | -0.2 (-1.3 to 1) |
| Anxiety *(0-24)* |  | -0.4 (-1.8 to 1) |  | -0.1 (-1.4 to 1.2) |
| Somatization *(0-32)* |  | -1.8 (-4 to 0.4) |  | -1.2 (-3.7 to 1.4) |
| Physical behaviour *(h/d)** |  |  |  |  |
| Sedentary behaviour |  | 0.3 (-0.8 to 1.5) |  | 0.5 (-0.5 to 1.5) |
| Moderate or vigorous physical activity |  | 0.1 (-0.2 to 0.4) |  | -0.1 (-0.4 to 0.1) |
| EQ VAS *(0-100)** |  |  |  |  |
| Overall current health |  | 5.8 (-2.3 to 13.8) |  | 5.4 (-2.4 to 13.2) |
| Illness perceptions IPQ-k *(0-10)** |  |  |  |  |
| Consequences |  | -0.1 (-1.4 to 1.2) |  | -0.2 (-1.4 to 0.9) |
| Timeline |  | 0 (-1.5 to 1.4) |  | 0.9 (-0.5 to 2.4) |
| Personal control |  | 1 (-0.4 to 2.4) |  | 0.6 (-0.6 to 1.8) |
| Treatment control |  | 1.1 (-0.5 to 2.7) |  | -0.1 (-1.6 to 1.3) |
| Identity |  | -0.3 (-1.3 to 0.6) |  | -0.7 (-1.8 to 0.3) |
| Concern |  | 0.2 (-1.2 to 1.6) |  | 0.2 (-1 to 1.4) |
| Coherence |  | 0.8 (-0.5 to 2.1) |  | 0.4 (-0.9 to 1.7) |
| Emotional response |  | 0.6 (-0.7 to 1.9) |  | 0.5 (-0.8 to 1.8) |
| Self-management skills HEI-Q *(1-4)** |  |  |  |  |
| Health-directed activity |  | 0.06 (-0.20 to 0.31) |  | -0.05 (-0.30 to 0.21) |
| Positive and active engagement in life |  | 0 (-0.21 to 0.21) |  | 0.13 (-0.10 to 0.36) |
| Self-monitoring and insight |  | 0.22 (0 to 0.45) |  | 0.22 (0.01 to 0.43) |
| Constructive attitude and approaches |  | -0.03 (-0.25 to 0.20) |  | 0.09 (-0.14 to 0.30) |
| Skill and technique acquisition |  | 0.21 (-0.13 to 0.54) |  | 0.18 (-0.06 to 0.41) |
| Social integration and support |  | 0.05 (-0.23 to 0.32) |  | -0.14 (-0.40 to 0.13) |
| Emotional distress |  | -0.01 (-0.25 to 0.23) |  | 0.01 (-0.26 to 0.27) |
| Health service navigation |  | 0.01 (-0.24 to 0.27) |  | -0.01 (-0.23 to 0.22) |
| *Data are differences in mean (95%CI).  †Data are odds ratio (95% CI).  Exp = experimental group (n=57), Con = control group (n=80) | | | | |
