# Supplementary material for: Effectiveness of a blended multidisciplinary intervention for patients with moderate medically unexplained physical symptoms (PARASOL): A cluster randomized clinical trial
Source: PLoS One. 2023 Apr 6;18(4):e0283162. doi: 10.1371/journal.pone.0283162 (PMC10079131; doi:10.1371/journal.pone.0283162)
Supplement: S1 File — (DOCX) [file pone.0283162.s003.docx]

S1 File. sensitivity analyses of subjective symptom impact

| At least 60% adequate relief on short-term | Groups | | Difference between groups |
| --- | --- | --- | --- |
|  | Exp | Con | Exp minus Con |
| Impact of symptoms Adequate relief *(yes/no)* |  |  |  |
| Responder, n(%) | 25 (31%) | 11 (14%) | OR 3.0  (1.2 to 7.6) |
| Exp = experimental group, Con = control group |  |  |  |

| At least 40% adequate relief on short-term | Groups | | Difference between groups |
| --- | --- | --- | --- |
|  | Exp | Con | Exp minus Con |
| Impact of symptoms Adequate relief *(yes/no)* |  |  |  |
| Responder, n(%) | 42 (53%) | 20 (25%) | OR 3.8  (1.7 to 8.2) |
| Exp = experimental group, Con = control group |  |  |  |

| At least 70% adequate relief on long-term | Groups | | Difference between groups |
| --- | --- | --- | --- |
|  | Exp | Con | Exp minus Con |
| Impact of symptoms Adequate relief *(yes/no)* |  |  |  |
| Responder, n(%) | 28 (35%) | 27 (34%) | OR 1.2  (0.5 to 3) |
| Exp = experimental group, Con = control group |  |  |  |

| At least 40% adequate relief on long-term | Groups | | Difference between groups |
| --- | --- | --- | --- |
|  | Exp | Con | Exp minus Con |
| Impact of symptoms Adequate relief *(yes/no)* |  |  |  |
| Responder, n(%) | 50 (63%) | 33 (41%) | OR 2.2  (1 to 4.8) |
| Exp = experimental group, Con = control group |  |  |  |
