# Supplementary material for: Effectiveness of a blended multidisciplinary intervention for patients with moderate medically unexplained physical symptoms (PARASOL): A cluster randomized clinical trial
Source: PLoS One. 2023 Apr 6;18(4):e0283162. doi: 10.1371/journal.pone.0283162 (PMC10079131; doi:10.1371/journal.pone.0283162)
Supplement: S2 File — (PDF) [file pone.0283162.s004.pdf]

Evaluation of a proactive preventive program in patients with medically unexplained physical symptoms

(May 2017)

- May 2015: adaptation section 11.5: text in accordance to old and new Measure regarding Compulsory Insurance for Clinical Research in Humans

PROTOCOL TITLE 'Evaluation of a proactive preventive program in patients with medically unexplained physical symptoms'

**Protocol ID**	***<include protocol ID given by sponsor or investigator>***
**Short title**	**Evaluation of the PARASOL intervention**
**EudraCT number**	**Not applicable**
**Version**	**3**
**Date**	**29/05/2017**
**Principal investigator**	**Prof. Dr. Cindy Veenhof**<br>**Dept. of Rehabilitation, Nursing Science and Sport**<br>**University Medical Centre Utrecht**<br>**C.Veenhof-2@umcutrecht.nl**<br>**Tel. (31) 88 75 60900**
**Coordinating investigator/project leader**	**Els van Westrienen, MSc, PT**<br>**PhD student**<br>**Dept. of Rehabilitation, Nursing Science and Sport**<br>**University Medical Centre Utrecht & Leidsche Rijn Julius Health Care Centers**<br>**p.e.vanwestrienen@umcutrecht.nl**<br>**tel. (31) 6 10055268**<br><br>**Dr. Martijn F. Pisters**<br>**Dept. of Rehabilitation, Nursing Science and Sport**<br>**University Medical Centre Utrecht & Leidsche Rijn Julius Health Care Centers**<br>**m.f.pisters@umcutrecht.nl**<br>**Tel. (31) 6 43042462**<br><br>**Prof. Dr. Niek J. de Wit**

	**Dept. of General Practice, Julius Center for Health Sciences and Primary Care, University Medical Centre Utrecht & Leidsche Rijn Julius Health Care Centers**<br>**N.J.deWit@umcutrecht.nl**<br>**Tel. (31) 88 75 68247**
Multicenter research: per site	**Leidsche Rijn Julius Gezondheidscentra**<br>**Prof. Dr. N.J. de Wit, medical director**<br>**Utrechtse Heuvelrug 130**<br>**3452 JA Vleuten**<br>**N.J.deWit@umcutrecht.nl**<br>**Tel. (31) 88 75 68247**<br><br>**Stichting Gezondheidscentra Eindhoven**<br>**Drs. E.R.J. Berends, member of Board of Directors**<br>**Postbus 8736**<br>**5605 LS Eindhoven**<br>**e.berends@sge.nl**<br>**Tel. (31) 40 71 16000**
**Sponsor**	**UMC Utrecht**<br>**Heidelberglaan 100**<br>**3584 CX Utrecht**<br>**088 755 5555**<br><br>**In collaboration with Fontys University of Applied Sciences, Leidsche Rijn Julius Gezondheidscentra and University of Applied**

	**Sciences Utrecht**
**Subsidising party**	**NWO**
**Independent expert**	**Dr. T.C. Olde Hartman, general practitioner**
**Laboratory sites**	**Not applicable**
**Pharmacy**	**Not applicable**

PROTOCOL SIGNATURE SHEET

Name	Signature	Date
**Head of Department:**<br>Prof. Dr. J.M.A. Visser-Meily<br>Dept. of Rehabilitation, Nursing Science and Sport<br>University Medical Centre Utrecht<br>J.M.A.Visser-Meily@umcutrecht.nl<br>Tel. (31) 88 755 5881		14/7/16
**Principal Investigator:**<br>Prof. Dr. Cindy Veenhof<br>Dept. of Rehabilitation, Nursing Science and Sport<br>University Medical Centre Utrecht<br>C.Veenhof-2@umcutrecht.nl<br>Tel. (31) 88 75 60900		**11/07/2016**

TABLE OF CONTENTS

1. INTRODUCTION AND RATIONALE 10
2. OBJECTIVES 11
3. STUDY DESIGN 11
Figure 1 Overview of the study 12
4. STUDY POPULATION 13
4.1 Population (base) 13
4.2 Inclusion criteria 13
4.3 Exclusion criteria 13
4.4 Sample size calculation 13
5. TREATMENT OF SUBJECTS 15
5.1 Investigational treatment 15
5.2 Use of co-intervention 16
5.3 Escape medication 16
6. INVESTIGATIONAL PRODUCT 17
7. NON-INVESTIGATIONAL PRODUCT 17
8. METHODS 17
8.1 Study parameters/endpoints 17
8.1.1 Main study parameter/endpoint 17
8.1.2 Secondary study parameters/endpoints 17
8.1.3 Other study parameters 18
8.2 Randomisation, blinding and treatment allocation 18
8.3 Study procedures 18
8.4 Withdrawal of individual subjects 21
8.4.1 Specific criteria for withdrawal 21
8.5 Replacement of individual subjects after withdrawal 22
8.6 Follow-up of subjects withdrawn from treatment 22
8.7 Premature termination of the study 22
9. SAFETY REPORTING 22
9.1 Section 10 WMO event 22
9.2 AEs, SAEs and SUSARs 22
9.2.1 Adverse events (AEs) 23
9.2.2 Serious adverse events (SAEs) 23
9.2.3 Suspected unexpected serious adverse reactions (SUSARs) 23
9.3 Annual safety report 23
9.4 Follow-up of adverse events 23
9.5 Data Safety Monitoring Board (DSMB) / Safety Committee 23
10. STATISTICAL ANALYSIS 23
10.1 Primary study parameter 23
10.2 Secondary study parameters 23
10.3 Other study parameters 24
10.4 Interim analysis 24

11. ETHICAL CONSIDERATIONS 24
11.1 Regulation statement 24
11.2 Recruitment and consent 24
11.3 Objection by minors or incapacitated subjects 25
11.4 Benefits and risks assessment, group relatedness........ 25
11.5 Compensation for injury 26
11.6 Incentives 26
12. ADMINISTRATIVE ASPECTS, MONITORING AND PUBLICATION........ 26
12.1 Handling and storage of data and documents........ 26
12.2 Monitoring and Quality Assurance 26
12.3 Amendments........ 27
12.4 Annual progress report........ 27
12.5 End of study report........ 27
12.6 Public disclosure and publication policy 27
13. STRUCTURED RISK ANALYSIS 27
14. REFERENCES 27
15. APPENDIX 1 32
16. APPENDIX 2 35

LIST OF ABBREVIATIONS AND RELEVANT DEFINITIONS

**ABR**	**ABR form, General Assessment and Registration form, is the application form that is required for submission to the accredited Ethics Committee (In Dutch, ABR = Algemene Beoordeling en Registratie)**
**AE**	**Adverse Event**
**AR**	**Adverse Reaction**
**CA**	**Competent Authority**
**CCMO**	**Central Committee on Research Involving Human Subjects; in Dutch: Centrale Commissie Mensgebonden Onderzoek**
**CV**	**Curriculum Vitae**
**DSMB**	**Data Safety Monitoring Board**
**EU**	**European Union**
**EudraCT**	**European drug regulatory affairs Clinical Trials**
**GCP**	**Good Clinical Practice**
**IB**	**Investigator's Brochure**
**IC**	**Informed Consent**
**IMP**	**Investigational Medicinal Product**
**IMPD**	**Investigational Medicinal Product Dossier**
**METC**	**Medical research ethics committee (MREC); in Dutch: medisch ethische toetsing commissie (METC)**
**(S)AE**	**(Serious) Adverse Event**
**SPC**	**Summary of Product Characteristics (in Dutch: officiële productinfomatie IB1-tekst)**
**Sponsor**	**The sponsor is the party that commissions the organisation or performance of the research, for example a pharmaceutical company, academic hospital, scientific organisation or investigator. A party that provides funding for a study but does not commission it is not regarded as the sponsor, but referred to as a subsidising party.**
**SUSAR**	**Suspected Unexpected Serious Adverse Reaction**
**Wbp**	**Personal Data Protection Act (in Dutch: Wet Bescherming Persoonsgevens)**
**WMO**	**Medical Research Involving Human Subjects Act (in Dutch: Wet Medisch-wetenschappelijk Onderzoek met Mensen**

SUMMARY

Rationale: Medically unexplained physical symptoms (MUPS) are a serious problem in primary care, with a spectrum from mild to moderate or chronic MUPS. The burden of chronic MUPS is substantial for patients, health care professionals and the society. Therefore, early identification of patients with moderate MUPS to prevent chronicity is needed. Recently a new screenings method with acceptable prognostic accuracy was developed using data from the electronic medical record of the general practitioner. Furthermore, we developed a proactive blended and multidisciplinary preventive intervention to reduce complaints of moderate MUPS and to prevent chronicity, called the PARASOL intervention.

Objective: The primary objective is to investigate the effectiveness of the PARASOL intervention on impact of symptoms and physical and mental dimensions of quality of life in patients with moderate MUPS compared with usual care.

Secondary objectives are to study:

- What is the influence of the PARASOL intervention on severity of symptoms, general health, physical behaviour, illness perception and self-efficacy in patients with moderate MUPS?
- What is the cost-effectiveness of the PARASOL intervention in patients with moderate MUPS?

Study design: Cluster randomized clinical trial

Study population: Patients with moderate medically unexplained physical symptoms, aged ≥ 18 years.

Intervention: The PARASOL intervention will take 12 weeks with 4-5 face to face sessions with the physical therapist and the mental health nurse as well as weekly online modules. The focus of the intervention is on modifiable prognostic risk factors of chronic MUPS, with integration of principles of the modified consequence model, central sensitisation, cognitive behavioural approach and graded activity.

Main study parameters/endpoints: The main study parameters are impact of symptoms and quality of life.

Nature and extent of the burden and risks associated with participation, benefit and group relatedness: The risks for subjects are expected to be negligible because of the low impact of the PARASOL intervention. The PARASOL intervention has five face-to-face contacts with the physical therapist, four with the mental health nurse and overarching online modules, with content according to the guidelines. Additionally, the burden of the measurements (filling in questionnaires) at baseline and after three and twelve months will take 30-45 minutes. Furthermore, participants have to wear an activity monitor at baseline, after three and twelve months for one week.

1. INTRODUCTION AND RATIONALE

Medically unexplained physical symptoms (MUPS) are a serious problem in primary care(1). Common unexplained symptoms in primary care include pain, fatigue, dizziness and general “malaise”(2). In the Dutch multidisciplinary guideline for MUPS and Somatoform Disorders, MUPS are defined as physical complaints that last for at least a few weeks and are not explained by a medical condition after proper medical examination(3,4). Of all complaints that patients present to their general practitioner (GP), 25–50% cannot be medically explained immediately(5). This percentage is also high in other health care settings. In the practice of the physical therapist, neurologist or internist, 40-60% of patient complaints can also not be explained after proper medical examination(6,7). MUPS can be regarded as a continuum with a spectrum from mild unexplained physical symptoms (low incidence, one or two domains, low impact) moderate symptomatology (more frequent, 2 or 3 domains, higher impact) and persisting or chronic MUPS (high impact, more clusters, chronic)(3,8,9). Seventy percent of the patients who consult their GP with a MUPS related diagnosis improve within two weeks (mild MUPS)(10-12). The other 30% of the patients still experience unexplained symptoms after three months (moderate MUPS)(12). The prevalence rate of patients with chronic MUPS in primary care is approximately 2.5%(5,9,12,13). Patients with chronic MUPS can be distinguished in patients with a functional somatic syndrome (FSS; such as fibromyalgia, chronic fatigue syndrome or irritable bowel syndrome) and patients with a somatic symptom disorder according to DSM V(14).

Despite its low prevalence, the burden of chronic MUPS is substantial(5). The impact on patients quality of life and daily functioning is high, with the lowest scores on domains physical functioning, pain, general health, vitality, and social functioning compared with patients with a major depressive disorder, patients with cancer and the general population(15,16). Patients with MUPS have an above average consultation rate(17), and are more subject to diagnostic procedures(10). For GPs, management of MUPS is challenging and often frustrating, due to the mismatch with the expectations of the patient(18). Besides frustration, GPs experience uncertainty and fear of missing a medical diagnosis, which results in overdiagnosis(10). Additionally, GPs do not timely recognize patients with MUPS(19). It takes about two years before MUPS is diagnosed. In those two years patients will see a medical specialist for about eight times, a physical therapist 14 times and a GP 15 times, leading to increased direct health care costs. Finally MUPS are also associated with increased indirect costs (e.g. work and insurance related costs)(17,20). Absenteeism occurs in approximately 39% of the patients with MUPS.

Treating patients with MUPS is complex. Many intervention studies were already conducted, evaluating pharmacological, psychological, exercise therapy and/or a combination of those. In these studies, the majority of the patients had chronic MUPS(21-24). Education about central sensitization, graded activity, cognitive behavioural therapy and relaxation exercises are known effective treatment modalities in patients with chronic MUPS(3,25). Little research is conducted in patients with moderate MUPS with the aim to prevent chronicity. This is partly

due to the fact that patients with moderate MUPS cannot adequately be identified. Early identification of patients with moderate MUPS will decrease the burden for patients, health care professionals and society. Recently, we developed a new screening method (PRESUME) to identify patients with moderate MUPS using data from the electronic medical record of the GP(26). Patients with moderate MUPS can be identified, the PRESUME screening method showed acceptable prognostic accuracy over 5 years follow-up(27). When patients with moderate MUPS can be early identified, those patients can receive a multidisciplinary primary care preventive intervention to reduce complaints of moderate MUPS and prevent chronicity. We developed a multidisciplinary blended primary care intervention (PARASOL), which is delivered in a combination of eHealth and face-to-face care. The expectation is that this blended care will promote self-management. However, the (cost)effectiveness of this PARASOL intervention needs to be established. It is hypothesised that the PARASOL intervention, focused on modifiable prognostic risk factors of chronic MUPS(1,3,27) in which principles of the modified consequence model(28), central sensitisation(25), cognitive behavioural approach(29-31) and graded activity(32-34) are integrated, can reduce impact and severity of symptoms and increase quality of life, general health, physical behaviour, illness perception and self-efficacy in patients with moderate MUPS.

2. OBJECTIVES

Primary Objective:
What is the effectiveness of the PARASOL intervention on impact of symptoms and physical and mental dimensions of quality of life in patients with moderate MUPS compared with usual care?

Secondary Objectives:

1. What is the influence of the PARASOL intervention on severity of symptoms, general health, physical behaviour, illness perception and self-efficacy in patients with moderate MUPS compared with usual care?
2. What is the cost-effectiveness of the PARASOL intervention in patients with moderate MUPS compared with usual care?

3. STUDY DESIGN

The effectiveness of the blended PARASOL intervention will be studied with a cluster randomized clinical trial with a three and twelve months follow-up period within a primary health care setting. Furthermore, cost-effectiveness will also be assessed six and nine months after baseline using a questionnaire that focus on health care use and indirect costs in the past three months.

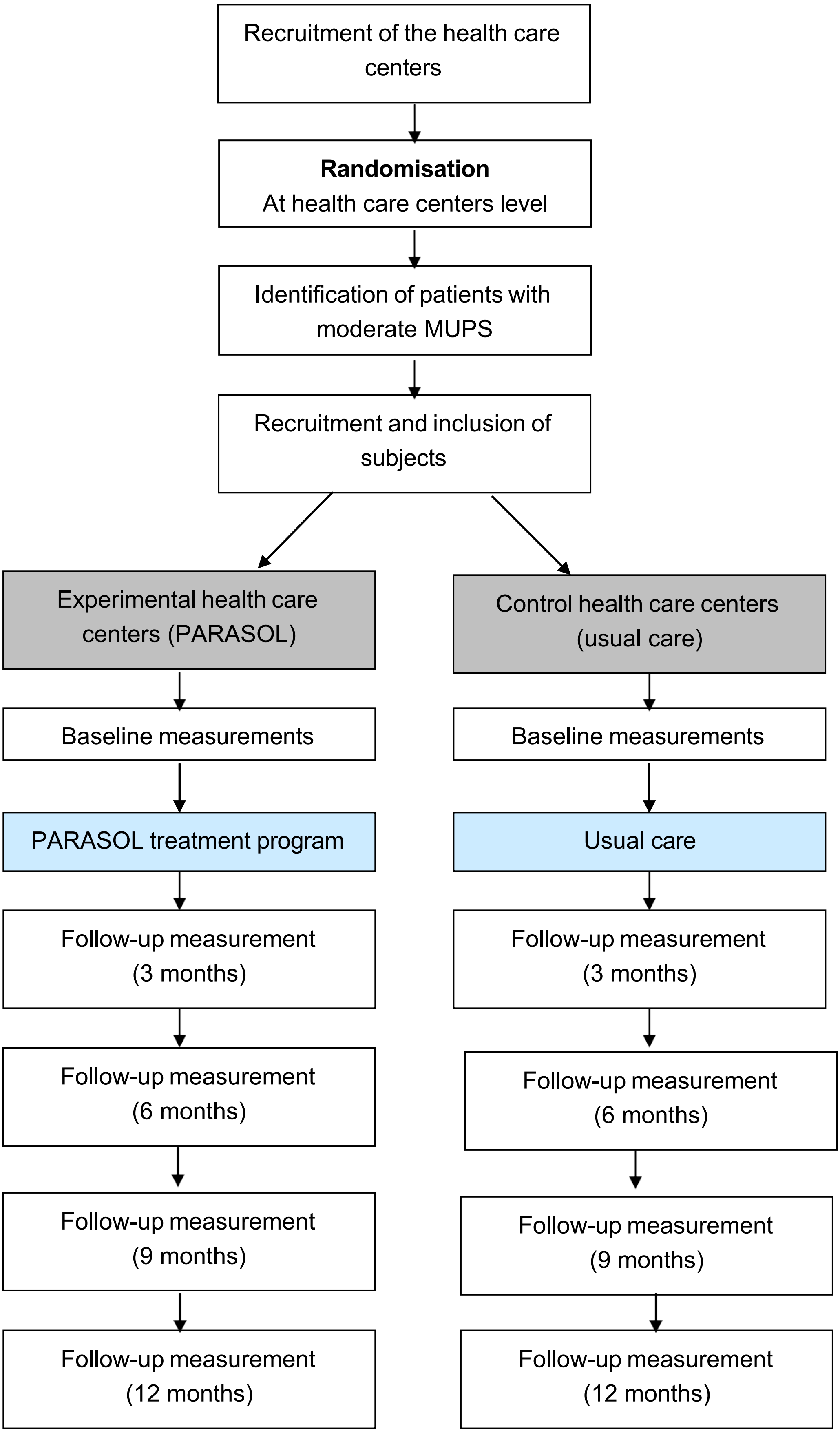

Figure 1 Overview of the study

4. STUDY POPULATION

4.1 Population (base)

In this study, 248 subjects with moderate MUPS from primary health care centers will be recruited.

4.2 Inclusion criteria

Subjects will be selected and identified with the PRESUME screening method (see figure 2).
In order to be eligible to participate in this study, a subject must meet all of the following criteria; the subjects have to:
- be 18 years or older
- not suffering from chronic somatic or psychiatric disease
- have five or more consultations with the GP in the past twelve months of which at least three of those consultations are with one of the 104 ICPC codes suggestive of MUPS (see Appendix 1).

Health care centers in primary care are eligible to participate if all relevant disciplines (general practitioner, physical therapist, mental health nurse) are available and willing to participate.

4.3 Exclusion criteria

Eligible subjects will be excluded when the subject has:
- insufficient mastering of the Dutch language
- no access to the internet.

Furthermore, all identified subjects with moderate MUPS using the PRESUME screening method will be screened by their GP. The GP will exclude the subjects if one or more of the following criteria apply:
- received a medical explained diagnosis between identification using the PRESUME method and the time of inclusion.
- complaints with a shorter duration than one month, in which more diagnostic evaluation of the symptoms is needed.
- unable to participate according to the GP, f.i. because of a life threatening condition, a shortened life expectancy, a major life event in the past month or a MUPS targeted multidisciplinary intervention in the past 12 months.
- the diagnostic phase has not been completed, in other words there is no agreement between GP and patient about sufficient diagnostic testing to rule out a physical cause

4.4 Sample size calculation

The number of eligible subjects was calculated according to Campbell et al. (2004) for cluster randomized trials(35). The total number of subjects depends on the mean cluster size and the correlation between the clusters, known as the intracluster (or intraclass)

correlation coefficient. The intracluster correlation coefficient is the proportion of the total variance on the outcome, explained by the variance between the clusters. The sample size needs to be corrected for the design effect, to calculate the power of the cluster randomized trials.

To calculate the sample size, we used an intracluster correlation coefficient of 0.04(36,37) and a minimum of 20 patients per health care center. Additionally, we assume a minimal clinical detectable change of >10 points in the sum score of physical functioning of the RAND-36 questionnaire(38), and a standard deviation of 23.8(16). Based on these assumptions and the assuming power of 80%, α=0.05, at least10 health care centers and overall 206 participating patients are needed. With an expected drop-out rate of 20% a total of 248 participating patients (per arm 124 patients) are needed.

The feasibility is checked with the average number of patients with moderate MUPS per primary care practice (54 patients; based on the numbers of Julius Primary Care Network) and the expected response (40%). Consequently, the estimation is that we can include 21 patients per health care center and at least 12 health care centers need to participate in the study to obtain the total number of participating patients. Co-applicants Leidsche Rijn Julius Gezondheidscentra and Stichting Gezondheidscentra Eindhoven (together 15 health care centers) are already willing to participate. Therefore, our expectation is that the feasibility is sufficient.

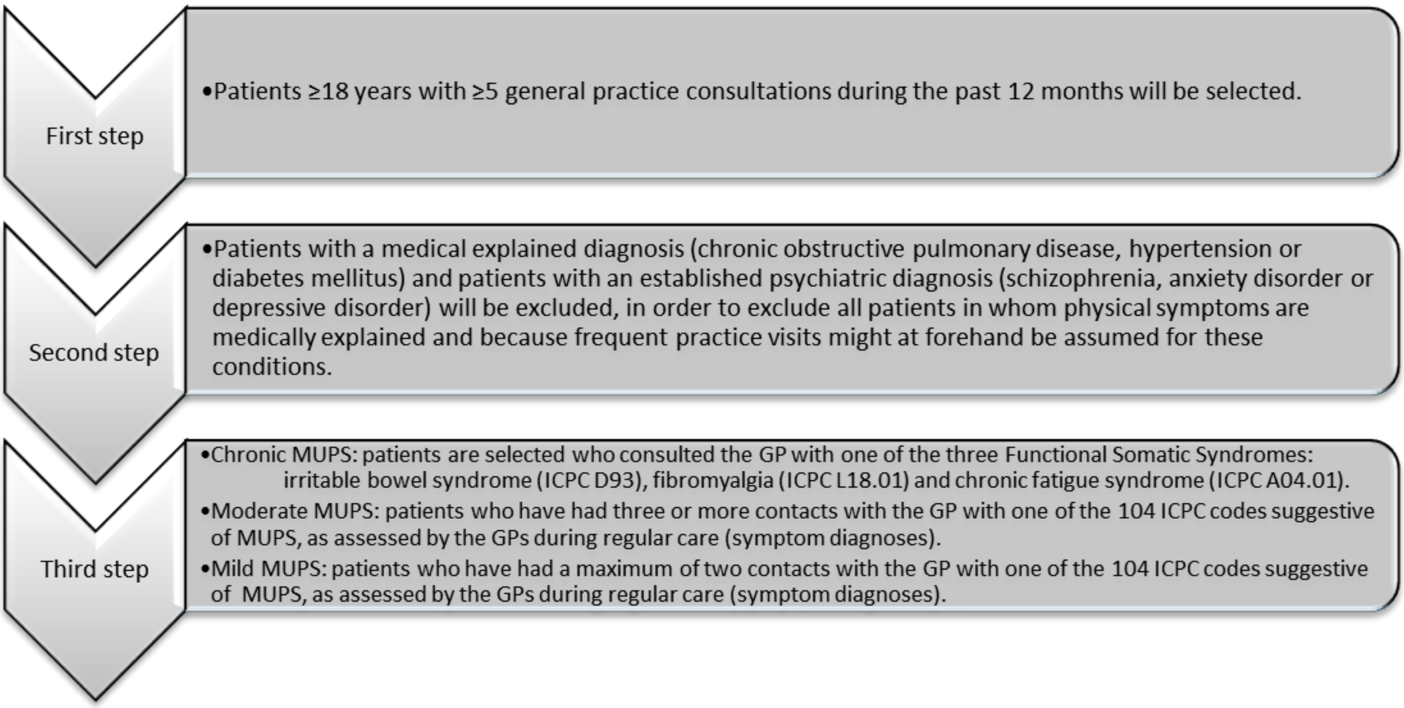

Figure 2 PRESUME screening method

5. TREATMENT OF SUBJECTS

The PARASOL intervention is a blended multidisciplinary primary care intervention of the physical therapist and mental health nurse. The intervention is developed according to the literature and focus groups with experts (general practitioners, physical therapists, mental health nurses and psychologists). The aim of the intervention is to decrease severity and impact of symptoms, to improve quality of life in patients with moderate MUPS and to prevent chronic MUPS by providing insight in factors that may adversely affect prognosis, encouraging self-management as well as an active lifestyle. The focus of the intervention is on modifiable prognostic risk factors for the development of chronic MUPS(1,3,27). The intervention is constructed according to the principles of the modified consequence model(28), central sensitisation(25), cognitive behavioural approach(29-31) and graded activity(32-34).

5.1 Investigational treatment

A. Active face-to-face treatment

The intervention will take 12 weeks with 8-10 treatment sessions, 4-5 sessions per discipline (intake, week 1, 3, 6 and 12). Both the physical therapist and the mental health nurse will have thirty minutes per session. During the intake at both health care professionals, the complaints, treatment demand and negative or maintaining factors of the patient will be identified according to the SCEGS(3). The physical therapist will start with focussing on physical functioning (exercise, stress, pain and fatigue). In the next step the mental health nurse will gradually incorporate psychological and social functioning in the treatment plan. Both health care professionals will integrate cognitive behavioural principles in their part of the intervention. The last session in week 12 will only be with the physical therapist. In this last session, the intervention will be evaluated and long-term goals will be discussed (see Appendix 2 for a schematic view of the PARASOL intervention).

A1 Physical therapy

The Dutch multidisciplinary guideline for MUPS recommends active physical therapy (e.g. exercise therapy)(3,4). In advance, we will start with education regarding the unexplained symptoms. Education according to the sensitisation model of Nijs and van Wilgen is of interest due to the somatic fixation and anxiety for a severe disease of the subject(25). Concurrently, graded activity will be used to expand activities gradually(32-34). The graded activity schedule can be performed in daily life. In this way, we will focus on

perception and acceptation of physical complaints of the subjects during the face-to-face sessions.

A 2 Mental health care

Besides physical therapy, the Dutch multidisciplinary guideline for MUPS and Somatoform Disorders and the MUPS standard of the Dutch college of general practitioners also recommend a referral to the mental health nurse(3,4). The mental health nurse can help the subjects in learning coping strategies according to the consequence model and operant conditioning, with the focus on changing perception and acceptation(39,40).

B. Self-management/Home exercises

The face-to-face intervention will be integrated with eHealth modules, called blended health care(41). With this blended health care, we will reduce the burden for subjects with less face-to-face sessions, promote self-management in the home environment for subjects and thus reduce health care costs. This blended health care is proven effective (42,43). The online part of the PARASOL intervention consists of exercises (instruction videos), information modules on self-management and educative themes (description and videos). The modules will consist of three components:

1. Graded activity, an activity-focused method with operant conditioning behavioural principles. The subject will perform a chosen activity to his/her tolerance level (i.e., until pain or fatigue drives them to stop; pain-contingent), while their performance is recorded in distance units or number of repetitions. After at least three measurements, occurring over several days, baseline is determined and the chosen activity will be increased gradually (time-contingent)(44).
2. Videos of advised exercises
3. Videos and information on self-management and educative themes: central sensitisation, the consequence model, graded activity, behavioral change, stress, coping, relaxation, lifestyle advises, creating and performing an exercise plan and avoiding a relapse.

The content used for each individual subject will be selected according to the complaints.

5.2 Use of co-intervention

Not applicable

5.3 Escape medication

Not applicable. Patients are able to continue their regular medication.

6. INVESTIGATIONAL PRODUCT

Not applicable.

7. NON-INVESTIGATIONAL PRODUCT

Not applicable

8. METHODS

8.1 Study parameters/endpoints

8.1.1 Main study parameter/endpoint

The primary parameters are adequate relief and quality of life. Adequate relief addresses symptoms improvement with a validated single question, scored on a dichotomous scale (“Over the past week have you had adequate relief of your symptoms?”)(45,46). A responder for adequate relief is defined as a subject who will report adequate relief of their symptoms for at least 6 of the 12 weeks between T0 and T1, and for at least 3 of the 6 months between T1 and T2. Otherwise, a subject will be defined as a non-responder. Adequate relief can be seen as a clinically relevant endpoint at which the individual subject is satisfied with treatment(47). Quality of life will be measured with RAND-36 health survey. The RAND-36 is a valid and reliable self-reported questionnaire. The questionnaire consists of eight subscales; physical functioning, social functioning, role-physical or emotional problems, mental health, vitality, bodily pain and general health. A higher score (0-100) indicate a better quality of life(48,49).

8.1.2 Secondary study parameters/endpoints

Different secondary parameters will be measured to determine the influence of the PARASOL intervention on general health, physical behaviour, perceptions, self-efficacy and cost-effectiveness.

- Severity of symptoms, defined as self-perceived pain and fatigue in the past week, will be measured with an 11-point numeric scale (score 0-10)(50).
- Severity of psychosocial symptoms will be measured with 4DKL questionnaire. This questionnaire consists of four subscales (distress, depression, anxiety, somatisation)(51,52).
- Health care use and indirect costs through illness and absenteeism will be measured with TIC-P questionnaire to evaluate cost-effectiveness for social perspective in terms of costs per ‘Quality Adjusted Life Years (QALYs)(53)’. QALYs will be measured using the SF-6D scores (according to SF-36 questionnaire)(53,54), which is a valid and reliable method(53).
- Physical behaviour will be measured with the Activ8 activity monitor(55). The Activ8 is a valid measurement in detecting lying, sitting, standing, walking, running and cycling. In this way, physical behaviour can be measured accurate. Subjects will wear the Activ8 activity monitor for one week. They can wear the Activ8 activity

monitor in their pants pocket without recharging it during the week. There are accessories as straps available in case subjects does not have pockets.

- Illness perceptions will be measured with the Brief Illness Perception Questionnaire. This questionnaire is an eight-item scale designed to assess cognitive and emotional representations of illness on an ordinal scale (0-10)(56,57).
- Self perceived health will be measured with the EQ5D questionnaire. This questionnaire will measure the perceived health on five levels (i.e. mobility, self-care, usual activities, pain/discomfort and anxiety/depression)(58).
- Self-efficacy will be measured with the Hei-Q questionnaire, which is a user friendly, valid and reliable questionnaire specifically developed in evaluating patients education and self-management programmes in patients with chronic complaints(59).

Besides the above parameters, efficacy, barriers and facilitators of the PARASOL intervention according to participating subjects and professionals will also be determined, using the 'System Usability Scale (SUS)'. The SUS will be completed at T1, at the end of the PARASOL intervention. The questionnaire will measure the perceived usability by ten statements which can be scored on a 5-point Likert scale ('totally agree' to 'totally disagree'). The SUS is a simple, valid and reliable measurement and is often used the evaluate the usability of eHealth applications(60).

8.1.3 Other study parameters

Demographic and clinical baseline variables such as age, gender, education level, work situation, duration of complaints and possible co-morbidities will be measured at baseline. Demographic and clinical variables will only be completed at baseline.

8.2 Randomisation, blinding and treatment allocation

We will recruit health care centers that fulfil the inclusion criterion. Co-applicants Leidsche Rijn Julius Gezondheidscentra (5 health care centers, 40.000 patients) and Stichting Gezondheidscentra Eindhoven (10 health care centers, 80.000 patients) are already willing to participate (co-applicants of the NWO grant proposal). The participating health care centers will be randomized to either the experimental group or the control group. Through cluster randomization, we will avoid the possibility of professionals offering both experimental and control intervention. In this way we also avoid that professionals who offer the experimental or control intervention within one health care center will influence each other (contamination-effect)(61). After randomization, the selection and inclusion procedure of subjects with moderate MUPS will be performed. In the health centers in the experimental group, patients with moderate MUPS will be offered the PARASOL intervention. In the control group health centers, patients with moderate MUPS will get care as usual.

8.3 Study procedures

Recruitment of subjects

After randomization of the health care centers, the health care centers will perform the PRESUME screening method using the electronic medical record of the GPs to identify subjects with moderate MUPS. The identified subjects will proactively be approached by the GP by sending them a letter as well as an information letter about the PARASOL study. The GP will send a reminder to the subjects who did not respond two weeks after sending the information letter. Another two weeks later, an assistant of the health care centers will contact the subjects by phone to see if they are willing to participate. When a subject is willing to participate, he can contact the researcher by phone or by mail. The researcher will answer possible questions, give further information, and will ask if the subject is willing to participate. When a subject is willing to participate, an appointment for signing informed consent will be made. After signing informed consent, baseline measurements will be completed (see patient report form) (see Chapter 8.1). After the baseline measurements, subjects of the experimental health care centers will be contacted by phone to make an appointment for the intake with the physical therapist and mental health nurse. Subjects of the control health care centers will be contacted three, six, nine and twelve months after completing baseline measurements for the follow-up measurements. In the meantime, they can receive care as usual (see figure 1). Subjects of the control health care centers will be offered the PARASOL intervention after the last follow-up measurement, twelve months after completing the baseline measurements. When their insurance does not cover the physical therapy part, the PARASOL intervention will be offered for free.

The total duration of the PARASOL study for all subjects (both experimental group and control group) will be twelve months. The PARASOL intervention for the subjects of the experimental group will start after the baseline measurements with an intake at the physical therapist and the mental health nurse and has a duration of twelve weeks.

Besides the above recruitment of subjects we will add two other recruitment strategies to increase the number of subjects. The first strategy is that GPs can recruit patients who are visiting the GP for consultation. The GP can recruit patients according to the following criteria:

- patients ≥ 18 years with ≥ 5 general practice consultations during the past twelve months
- GP cannot medically explain the symptoms
- GP already completed the diagnostic phase in accordance with the patient

When a patient is eligible, the GP can give the patient the information letter about the PARASOL study. In addition, the GP will ask if the researchers can contact the patient by phone. If the patient agree, the GP will inform the researchers and give the name and phone number of the patient.

A second strategy will be an open recruitment in the participating health care centers. Flyers with information of the PARASOL study will be laid down in the waiting rooms. In addition, the flyer will be added in the newsletter of Leidsche Rijn Julius Gezondheidscentra as well as the newsletter of Stichting Gezondheidscentra

Eindhoven and will be send to all patients by mail. Patients who are willing to participate can contact the researcher by phone or by mail. Subsequently, the researcher will contact the GP to check eligibility of the patient according to the above criteria.

Besides the two new recruitment strategies, the GPs in the participating health care centers mentioned that patients had difficulties in understanding the information letter. Therefore, we would like to add the flyer besides sending the letter of the GP and the information letter about the PARASOL study for the remaining 520 patients identified by the PRESUME screening method and selected by the GP.

The researcher will contact the subject by phone to make an appointment for the baseline assessment which will be performed by subjects at home. At the baseline assessment patient report form A will be completed, subjects will be informed and instructed about wearing the Activ8 activity monitor and patient report form B. Subjects will be asked to carry the Activ8 for one week to measure their physical behaviour as well as requested to complete patient report form B digitally. After one week, they can send the Activ8 back by mail. Subjects are asked to complete the adequate relief question weekly between T0 and T1.
The first follow-up measurement (T1) will be three months after baseline. Subjects will be asked to complete the follow-up measurements and to carry the Activ8 for one week.
Six months and nine months after baseline, subjects will be asked to complete the cost-effectiveness questionnaire, since this questionnaire focuses on health related costs and indirect costs of the past three months. Additionally, subjects are asked to complete the adequate relief question monthly six months after baseline until T2.
The last follow-up measurement (T2) will be twelve months after baseline. Subjects will be asked to complete the follow-up measurements and to carry the Activ8 for one week. Table 1 gives a summary of all measures that will be collected with the different collection points.

Table 1: Summary of measures to be collected

**Primary outcome measures**	**Data collection instrument**	**Follow-up measurements**				
		**Baseline**	**3 mo.**	**6 mo.**	**9 mo.**	**12 mo.**
Impact of symptoms	Adequate Relief question	X	X			X
		Weekly between baseline and 3 months after baseline		Monthly between six and twelve months after baseline		
Quality of life	36-Item Short Form Health Survey (RAND-36)	X	X			X
**Secondary outcome measures**						
Pain	Numeric Rating Scale (NRS)	X	X			X

Fatigue	Numeric Rating Scale (NRS)	X	X			X
Severity of psychosocial symptoms	Four-Dimensional Symptom Questionnaire (4DKL)	X	X			X
General health	EuroQol - 5 Dimensions (EQ5D)	X	X			X
Physical behaviour	Activ8 activity monitor	X	X			X
Illness perceptions	Brief Illness Perception Questionnaire (IPQ-K)	X	X			X
Self-efficacy	Health Education Impact Questionnaire (Hei-Q)	X	X			X
Cost-effectiveness	Trimbos and iMTA questionnaire on Costs associated with Psychiatric illness (TIC-P)	X	X	X	X	X
Barriers and facilitators of the PARASOL intervention	System Usability Scale (SUS)		X			
**Other measures**						
Age	Questionnaire	X				
Gender	Questionnaire	X				
Education level	Questionnaire	X				
Work situation	Questionnaire	X				
Duration of complaints	Questionnaire	X				
Possible co-morbidities	Questionnaire	X				

<u>PARASOL intervention</u>

As written in Chapter 5 the PARASOL intervention will consist of face-to-face appointments as well as online modules. The PARASOL intervention will take 12 weeks with 4-5 face to face sessions with the physical therapist as well as with the mental health nurse and weekly new online modules (see Figure 3 and appendix 2).

<u>Usual care</u>

Subjects of the control health care centers will get care as usual without any restrictions. They can still receive care (e.g. diagnostic procedures or interventions) of the GP or any other health care professional, including a physical therapist or mental health nurse.

8.4 Withdrawal of individual subjects

Subjects can leave the study at any time for any reason if they wish to do so without any consequences. The investigator can decide to withdraw a subject from the study for urgent medical reasons.

8.4.1 Specific criteria for withdrawal

Not applicable

8.5 Replacement of individual subjects after withdrawal

Subjects who withdraw will not be replaced.

8.6 Follow-up of subjects withdrawn from treatment

Subjects who withdraw from treatment will be asked to complete the follow-up measurements.

8.7 Premature termination of the study

When serious adverse events will be recorded that influence the health of the subjects, we will terminated the study prematurely (see Chapter 9).

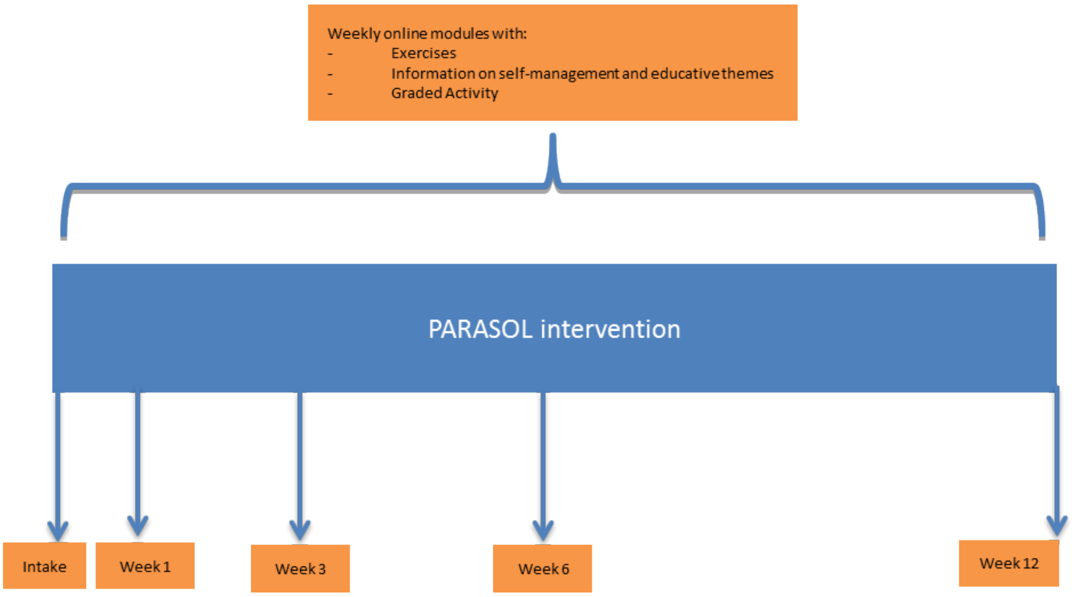

Figure 3 Overview of the PARASOL intervention

9. SAFETY REPORTING

9.1 Section 10 WMO event

In accordance to section 10, subsection 4, of the WMO, the sponsor will suspend the study if there is sufficient ground that continuation of the study will jeopardise subject health or safety. The sponsor will notify the accredited METC without undue delay of a temporary halt including the reason for such an action. The study will be suspended pending a further positive decision by the accredited METC. The investigator will take care that all subjects are kept informed.

9.2 AEs, SAEs and SUSARs

The expectation is that the PARASOL intervention will not lead to (serious) adverse events (AE's or SAE's). However, in case of (serious) adverse events, we will follow the normal procedure as mentioned below.

9.2.1 Adverse events (AEs)

Adverse events are defined as any undesirable experience occurring to a subject during the study, whether or not considered related to PARASOL intervention. All adverse events reported spontaneously by the subject or observed by the investigator or his staff will be recorded.

9.2.2 Serious adverse events (SAEs)

The sponsor will report the SAEs through yearly line-listings to the accredited METC that approved the protocol.

9.2.3 Suspected unexpected serious adverse reactions (SUSARs)

Not applicable

9.3 Annual safety report

Not applicable

9.4 Follow-up of adverse events

All AEs will be followed until they have abated, or until a stable situation has been reached. Depending on the event, follow up may require additional tests or medical procedures as indicated, and/or referral to the general physician or a medical specialist. SAEs need to be reported till end of study within the Netherlands, as defined in the protocol.

9.5 Data Safety Monitoring Board (DSMB) / Safety Committee

Not applicable. After consulting the secretary of the internal DSMB, we concluded that due to the low risk of the study an internal DSMB is not indicated.

10. STATISTICAL ANALYSIS

Statistical analysis will be performed with IBM SPSS 22. Statistical analysis will be performed according to the ‘intention-to-treat’ principle. Any missing values will be imputed with the ‘Multivariate Imputation by Chained Equations’.

10.1 Primary study parameter

Differences in effectiveness of the PARASOL intervention will be analysed using longitudinal mixed methods analyses. In this way, we can correct for independence of observations within subjects as well as take into account possible variations between clusters and health care professionals. Analyses will be corrected for potential confounders.

10.2 Secondary study parameters

Statistical analysis of the primary study parameter will also be performed for the secondary study parameters. Furthermore, cost-effectiveness of the PARASOL intervention will be clarified with an incremental cost-effectiveness ratio (ICER) based on

the costs per ‘quality adjusted life year (QALY)’. All costs measured by the TIC-P (health care use and indirect costs of illness and absenteeism) are used to calculate the ICER.

10.3 Other study parameters

The demographic and clinical baseline variables will be compared with t-tests and chi-square analyses to define if we included two comparable groups. Additionally, means and standard deviation will be calculated.

10.4 Interim analysis

Not applicable.

11. ETHICAL CONSIDERATIONS

11.1 Regulation statement

This study will be conducted according to the principles of the Declaration of Helsinki (version 2013) and in accordance with the Medical Research Involving Human Subjects Act (WMO).

11.2 Recruitment and consent

After randomization of the health care centers, the health care centers will perform the PRESUME screening method using the electronic medical record of the GPs to identify subjects with moderate MUPS. The identified subjects will proactively be approached by the GP by sending them a letter as well as an information letter about the PARASOL study. When the subject is willing to participate, he can contact the researcher by phone or by mail. The researcher will answer all possible questions and give further information. Otherwise, a reminder will be send two weeks after sending the information letter. Another two weeks later, an assistant of the health care center will contact the subjects by phone to see if they are willing to participate. Informed consent will be signed before completing baseline measurements. After completing baseline measurements, subjects of the experimental health care centers will be contacted by phone to make an appointment for the intake with the physical therapist and mental health nurse. Subjects of the control health care centers will be contacted three, six, nine and twelve months after completing baseline measurements for the follow-up measurements. In the meantime, they can receive care as usual (see figure 1). Subjects of the control health care centers will be offered the PARASOL intervention after the last follow-up measurement, twelve months after completing the baseline measurements. When their insurance is insufficient, the PARASOL intervention will be offered for free.
Besides the above recruitment of subjects we will add two other recruitment strategies to increase the number of subjects. The first strategy is that GPs can recruit patients who are visiting the GP for consultation. The GP can recruit patients according to the following criteria:
- patients ≥ 18 years with ≥ 5 general practice consultations during the past twelve months

- GP cannot medically explain the symptoms
- GP already completed the diagnostic phase in accordance with the patient

When a patient is eligible, the GP can give the patient the information letter about the PARASOL study. In addition, the GP will ask if the researchers can contact the patient by phone. If the patient agree, the GP will inform the researchers and give the name and phone number of the patient.
A second strategy will be an open recruitment in the participating health care centers. Flyers with information of the PARASOL study will be laid down in the waiting rooms. In addition, the flyer will be added in the newsletter of Leidsche Rijn Julius Gezondheidscentra as well as the newsletter of Stichting Gezondheidscentra Eindhoven and will be send to all patients by mail. Patients who are willing to participate can contact the researcher by phone or by mail. Subsequently, the researcher will contact the GP to check eligibility of the patient according to the above criteria.

Besides the two new recruitment strategies, the GPs in the participating health care centers mentioned that patients had difficulties in understanding the information letter. Therefore, we would like to add the flyer besides sending the letter of the GP and the information letter about the PARASOL study for the remaining 520 patients identified by the PRESUME screening method and selected by the GP.

11.3 Objection by minors or incapacitated subjects

Not applicable

11.4 Benefits and risks assessment, group relatedness

<u>Benefits:</u>

Subjects randomized in the PARASOL intervention can have beneficial effects of the intervention on their experienced quality of life, severity of symptoms and adequate relief. There are no benefits of participation during the study for subjects randomized in the control group. However, subjects of the control group will be offered the complete PARASOL intervention after the last follow-up measurement, twelve months after completing the baseline measurements.

<u>Risks:</u>

The study is carried out with adults. The risks for the subjects are minimal because of the low burden of the intervention for the subjects in the experimental group. The focus in the PARASOL intervention will be on modifiable prognostic risk factor of chronic MUPS through exercise therapy, coaching using face-to-face contact and an eHealth module, based on current literature, guidelines and focus groups with experts. Additionally, there are no risks for the subjects in the control group, since they will receive care as usual.

The duration of the sessions with the physical therapist and mental health nurse will take 30 minutes per session. The duration of the online part of the PARASOL intervention will take one hour weekly. Filling in questionnaires at baseline, after three and twelve months will take 30-45 minutes. In addition, subjects will complete the questionnaire on health care use and indirect costs six and nine months after baseline, which will take approximately 15 minutes. The adequate relief question will be completed more often and will take a maximum of 5 minutes each time. The adequate relief question will be completed weekly between T0 and T1, and monthly between six and twelve months after baseline. Additionally, subjects have to wear the Activ8 activity monitor at baseline, after three and twelve months for one week. Wearing the Activ8 activity monitor has a low burden since the Activ8 can be worn in the pants pocket and does not have to be recharged during the week. There are accessories as straps available in case subjects does not have pockets.

11.5 Compensation for injury

The sponsor/investigator has a liability insurance which is in accordance with article 7 of the WMO.

The sponsor/investigator has requested dispensation for the subject insurance because participating in the study is with low risk.

11.6 Incentives

Not applicable. Subjects will get a report about their individual progress on the primary outcome measures as well as on their physical behaviour after their last follow-up measurement. Additionally, they will get a report on their progress compared to the other subjects at the end of the study.

12. ADMINISTRATIVE ASPECTS, MONITORING AND PUBLICATION

12.1 Handling and storage of data and documents

Data will be handled confidentially. Subjects will be coded by numbers, and the code list will only be accessible by the principle investigators. Source data will be stored on the secured network of the University Medical Centre Utrecht and will only be accessible by the principle investigators. The guidelines for data handling and storage will be followed.

12.2 Monitoring and Quality Assurance

Our study will be monitored by a central internal monitor of the Julius Center at UMC Utrecht. There will be one initiation visit and close-out visit per primary health care setting. The monitoring visits will be once per year. Rates of inclusion and drop-outs, study files,

informed consents, in- and exclusion criteria, source data verification, serious adverse events and research procedures will be monitored. Details of the monitoring are described in our monitoring plan.

12.3 Amendments

All substantial amendments will be notified to the METC and to the competent authority. Non-substantial amendments will not be notified to the accredited METC and the competent authority, but will be recorded and filed by the sponsor.

12.4 Annual progress report

The sponsor/investigator will submit a summary of the progress of the trial to the accredited METC once a year. Information will be provided on the date of inclusion of the first subject, numbers of subjects included and numbers of subjects that have completed the trial, serious adverse events/ serious adverse reactions, other problems, and amendments.

12.5 End of study report

The investigator will notify the accredited METC of the end of the study within a period of 8 weeks. The end of the study is defined as the last patient's follow-up measurement.

In case the study is ended prematurely, the investigator will notify the accredited METC within 15 days, including the reasons for the premature termination.

Within one year after the end of the study, the investigator/sponsor will submit a final study report with the results of the study, including any publications/abstracts of the study, to the accredited METC.

12.6 Public disclosure and publication policy

The study is sponsored by SIA RAAK publiek (NWO). The arrangements with the sponsor is such that there will be no involvement on any kind of the sponsor in the public disclosure of results.

13. STRUCTURED RISK ANALYSIS

Not applicable

14. REFERENCES

(1) olde Hartman TC, Borghuis MS, Lucassen PL, van de Laar FA, Speckens AE, van Weel C. Medically unexplained symptoms, somatisation disorder and hypochondriasis: course and prognosis. A systematic review. J Psychosom Res 2009;May;66(5):363-77.

(2) Brown RJ. Introduction to the special issue on medically unexplained symptoms: background and future directions. Clin Psychol Rev 2007 Oct;27(7):269-80.

(3) Landelijke Stuurgroep Multidisciplinaire Richtlijnontwikkeling in de GGZ. Multidisciplinaire Richtlijn Somatisch Onvoldoende Verklaarde Lichamelijke Klachten En Somatoforme Stoornissen (Multidisciplinary Guideline of MUPS and Somatoform Disorders). Utrecht the Netherlands Trimbos Instituut;2010.

(4) Olde Hartman TC, Blankenstein AH, Molenaar AO, et al. NHG-Standaard Somatisch Onvoldoende verklaarde Lichamelijk Klachten (SOLK). Huisarts Wet 2013 56;5:222-30.

(5) Verhaak PF, Meijer SA, Visser AP, Wolters G. Persistent presentation of medically unexplained symptoms in general practice. Fam Pract 2006 aug;23(4):414-20.

(6) Nimnuan C, Hotopf M, Wessely S. Medically unexplained symptoms: an epidemiological study in seven specialities. J Psychosom Res 2001 Jul;51(1):361-7.

(7) Speckens AE, van Hemert AM, Spinhoven P, Hawton KE, Bolk JH, Rooijmans HG. Cognitive behavioural therapy for medically unexplained physical symptoms: a randomised controlled trial. BMJ 1995 Nov;18(311(7016)):1328-32.

(8) Smith RC DF. Classification and diagnosis of patients with medically unexplained symptoms. J Gen Intern Med 2007 May;22(5):685-91.

(9) Swanson LM, Hamilton JC, Feldman MD. Physician-based estimates of medically unexplained symptoms: a comparison of four case definitions. Fam Pract 2010 Oct;27(5):487-93.

(10) Kroenke K JJ. Outcome in general medical patients presenting with common symptoms: a prospective study with a 2-week and a 3-month follow-up. Fam Pract 1998 Oct;15(5):398-403.

(11) Kroenke K, Spitzer RL, Williams JB. The PHQ-15: validity of a new measure for evaluating the severity of somatic symptoms. Psychosom Med 2002 Mar-Apr;64(2):258-66.

(12) Jackson JL PM. The outcomes among patients presenting in primary care with a physical symptom at 5 years. J Gen Intern Med 2005 Nov;20(11):1032-7.

(13) Aamland A, Malterud K, Werner EL. Patients with persistent medically unexplained physical symptoms: a descriptive study from Norwegian general practice. BMC Fam Pract 2014 May;29(15):107.

(14) American Psychiatric Association.
Diagnostic and statistical manual of mental disorders (DSM-5). 5th ed. Washington: American Psychiatric Press; 2013.

(15) Koch H, van Bokhoven MA, ter Riet G, van der Weijden T, Dinant GJ, Bindels PJ. Demographic characteristics and quality of life of patients with unexplained complaints: a descriptive study in general practice. Qual Life Res 2007 Nov;16(9):1483-9.

(16) Zonneveld LN, Sprangers MA, Kooiman CG, van 't Spijker A, Busschbach JJ. Patients with unexplained physical symptoms have poorer quality of life and higher costs than other patient groups: a cross-sectional study on burden. BMC Health Serv Res 2013 Dec;17(13):520.

(17) Khan AA, Khan A, Harezlak J, Tu W, Kroenke K. Somatic symptoms in primary care: etiology and outcome. Psychosomatics 2003 Nov-Dec;44(6):471-8.

(18) Isaac ML PD. Medically unexplained symptoms. Med Clin North Am 2014 May;98(3):663-72.

(19) Olde Hartman T, Hassink-Franke L, Dowrick C, Fortes S, Lam C, van der Horst H, et al. Medically unexplained symptoms in family medicine: defining a research agenda. Proceedings from WONCA 2007 . Fam Pract 2008 Aug;25(4):266-71.

(20) Konnopka A, Schaefert R, Heinrich S, Kaufmann C, Luppa M, Herzog W, König HH. Economics of medically unexplained symptoms: a systematic review of the literature. Psychother Psychosom 2012 81;5:265-75.

(21) Hoedeman R, Blankenstein AH, van der Feltz-Cornelis CM, Krol B, Stewart R, Groothoff JW. Consultation letters for medically unexplained physical symptoms in primary care. Cochrane Database Syst Rev 2010 Dec;8(12):CD006524.

(22) Kleinstäuber M, Witthöft M, Steffanowski A, van Marwijk H, Hiller W, Lambert MJ. Pharmacological interventions for somatoform disorders in adults. Cochrane Database Syst Rev 2014 Nov;7(11).

(23) Rosendal M, Blankenstein AH, Morriss R, Fink P, Sharpe M, Burton C. Enhanced care by generalists for functional somatic symptoms and disorders in primary care. Cochrane Database Syst Rev 2013 Oct;18(10).

(24) van Dessel N, den Boeft M, van der Wouden JC, Kleinstäuber M, Leone SS, Terluin B, Numans ME, van der Horst HE, van Marwijk H. Non-pharmacological interventions for somatoform disorders and medically unexplained physical symptoms (MUPS) in adults. Cochrane Database Syst Rev 2014 Nov;1(11).

(25) Nijs J, van Wilgen P, Van Oosterwijck J, van Ittersum M, Meeus M. How to explain central sensitization to patients with 'unexplained' chronic musculoskeletal pain: practice guidelines. Man Ther 2011 Oct;16(5):413-8.

(26) van Westrienen PE, Rydell-Lexmond T, Pisters MF, Veenhof C, den Boeft M, Numans ME, et al. Identification of patients at risk for Medically Unexplained Physical Symptoms using primary care registration data; classification and patient characteristics

 .

(27) van Westrienen PE, Pisters MF, Veenhof C, de Wit NJ. Identification of patients at risk for medically unexplained physical symptoms in primary care with a five years follow-up
 .

(28) Zonneveld LN, Duivenvoorden HJ, Passchier J, van 't Spijker A. Tailoring a cognitive behavioural model for unexplained physical symptoms to patient's perspective: a bottom-up approach. Clin Psychol Psychother 2010 Nov-Dec;17(6):528-35.

(29) Linton SJ, & Nordin E. A 5-year follow-up evaluation of the health and economic consequences of an early cognitive behavioral intervention for back pain: a randomized, controlled trial. Spine (Phila Pa 1976) 2006 Apr;15(31(8)):853-8.

(30) Linton SJ AT. Can chronic disability be prevented? A randomized trial of a cognitive-behavior intervention and two forms of information for patients with spinal pain. Spine (Phila Pa 1976) 2000 Nov;25(21):2825-31.

(31) Linton SJ RM. A cognitive-behavioral group intervention as prevention for persistent neck and back pain in a non-patient population: a randomized controlled trial. Pain 2001 Feb 1;90(1-2):83-90.

(32) Fordyce WE, Fowler RS Jr, Lehmann JF, Delateur BJ, Sand PL, Trieschmann RB. Operant conditioning in the treatment of chronic pain. Arch Phys Med Rehabil 1973 Sep;54(9):399-408.

(33) Lindström I, Ohlund C, Eek C, Wallin L, Peterson LE, Fordyce WE, et al. The effect of graded activity on patients with subacute low back pain: a randomized prospective clinical study with an operant-conditioning behavioral approach. Phys Ther 1992 Apr;72(4):279-93.

(34) Pisters MF, Veenhof C, de Bakker DH, Schellevis FG, Dekker J. Behavioural graded activity results in better exercise adherence and more physical activity than usual care in people with osteoarthritis: a cluster-randomised trial. J Physiother 2010;56(1):41-7.

(35) Campbell MK, Elbourne DR, Altman DG, CONSORT group. CONSORT statement: extension to cluster randomised trials. BMJ 2004 Mar;20(328(7441)):702-8.

(36) Eccles M, Grimshaw J, Steen N, Parkin D, Purves I, McColl E, et al. The design and analysis of a randomized controlled trial to evaluate computerized decision support in primary care: the COGENT study. Fam Pract 2000 Apr;17(2):180-6.

(37) Thomas RE, Grimshaw JM, McClinton S, McIntosh E, Mollison J, Deans H, et al. An Evaluation of a guideline-based open access urological investigation service (URGE). Final project report to the Chief Scientist Office of the Scottish Home and Health Department
. Health Services Research Unit, Univerity of Aberdeen 1998.

(38) Jason LA, Taylor RR, Kennedy CL. Chronic fatigue syndrome, fibromyalgia, and multiple chemical sensitivities in a community-based sample of persons with chronic fatigue syndrome-like symptoms. Psychosom Med 2000 Sep-Oct;62(5):655-63.

(39) Wilkinson P ML. Problem-solving therapy in the treatment of unexplained physical symptoms in primary care: a preliminary study. J Psychosom Res 1994 Aug;38(6):591-8.

(40) Brunner E, De Herdt A, Minguet P, Baldew SS, Probst M. Can cognitive behavioural therapy based strategies be integrated into physiotherapy for the prevention of chronic low back pain? A systematic review. Disabil Rehabil 2013 Jan;35(1):1-10.

(41) Kelders SM, Kok RN, Ossebaard HC, Van Gemert-Pijnen JE. Persuasive system design does matter: a systematic review of adherence to web-based interventions. J Med Internet Res 2012 Nov;14(14(6)).

(42) Zeylemaker MM, Linn FH, Vermetten E. Blended care; development of a day treatment program for medically unexplained physical symptoms (MUPS) in the Dutch Armed Forces. Work 2015 50;1:111-20.

(43) Volker D, Zijlstra-Vlasveld MC, Anema JR, Beekman AT, Brouwers EP, Emons WH, et al. Effectiveness of a blended web-based intervention on return to work for sick-listed employees with common mental disorders: results of a cluster randomized controlled trial. J Med Internet Res 2015 May;13(17(5)).

(44) Gatzounis R, Schrooten MG, Crombez G, Vlaeyen JW. Operant learning theory in pain and chronic pain rehabilitation. Curr Pain Headache Rep 2012 Apr;16(2):117-26.

(45) Bijkerk CJ, de Wit NJ, Muris JW, Jones RH, Knottnerus JA, Hoes AW. Outcome measures in irritable bowel syndrome: comparison of psychometric and methodological characteristics. Am J Gastroenterol 2003 Jan;98(1):122-7.

(46) Passos MC, Lembo AJ, Conboy LA, Kaptchuk TJ, Kelly JM, Quilty MT, et al. Adequate relief in a treatment trial with IBS patients: a prospective assessment. Am J Gastroenterol 2009 Apr;104(4):912-9.

(47) Mangel AW, Hahn BA, Heath AT, Northcutt AR, Kong S, Dukes GE, et al. Adequate relief as an endpoint in clinical trials in irritable bowel syndrome. J Int Med Res 1998 Mar-Apr;26(2):76-81.

(48) Van der Zee KI, Sanderman R, Heyink JW, de Haes H. Psychometric qualities of the RAND 36-Item Health Survey 1.0: a multidimensional measure of general health status. Int J Behav Med 1996;3(2):104-22.

(49) Van der Zee KI, Sanderman R. Het meten van de algemene gezondheidstoestandmet de RAND-36, een handleiding (Measurement of general healthwith the RAND-36, amanual). Groningen, the Netherlands: Noordelijk Centrum voor Gezondheidsvraagstukken; 1993.

(50) Dworkin RH, Turk DC, Wyrwich KW, Beaton D, Cleeland CS, Farrar JT, et al. Interpreting the clinical importance of treatment outcomes in chronic pain clinical trials: IMMPACT recommendations. J Pain 2008 Feb;9(2):105-21.

(51) Terluin B, van Marwijk HW, Adèr HJ, de Vet HC, Penninx BW, Hermens ML, et al. The Four-Dimensional Symptom Questionnaire (4DSQ): a validation study of a multidimensional self-report questionnaire to assess distress, depression, anxiety and somatization. BMC Psychiatry 2006 Aug;22(6):34.

(52) Terluin B. De Vierdimensionale Klachtenlijst (4DKL): Een vragenlijst voor het meten van distress, depressie, angst en somatisatie. Huisarts Wet 1996;39:538-47.

(53) Bouwmans C, De Jong K, Timman R, Zijlstra-Vlasveld M, Van der Feltz-Cornelis C, Tan Swan S, et al. Feasibility, reliability and validity of a questionnaire on healthcare consumption and productivity loss in patients with a psychiatric disorder (TiC-P). BMC Health Serv Res 2013 Jun;15(13):217.

(54) Brazier J, Roberts J, Deverill M. The estimation of a preference-based measure of health from the SF-36. J Health Econ 2002 Mar;21(2):271-92.

(55) Bussmann H. Validation of the Active8 Activity Monitor: detection of body postures and movements. Publisher: Erasmus MC, Rotterdam, the Netherlands 2013.

(56) Broadbent E, Petrie KJ, Main J, Weinman J. The brief illness perception questionnaire. J Psychosom Res 2006 Jun;60(6):631-7.

(57) Leysen M, Nijs J, Meeus M, Paul van Wilgen C, Struyf F, Vermandel A, et al. Clinimetric properties of illness perception questionnaire revised (IPQ-R) and brief illness perception questionnaire (Brief IPQ) in patients with musculoskeletal disorders: A systematic review. Man Ther 2015 Feb;20(1):10-7.

(58) The EuroQol Group. EuroQol-a new facility for the measurement of health-related quality of life. Health Policy 1990 Dec;16(3):199-208.

(59) Osborne RH, Elsworth GR, Whitfield K. The Health Education Impact Questionnaire (heiQ): an outcomes and evaluation measure for patient education and self-management interventions for people with chronic conditions. Patient Educ Couns 2007 May;66(2):192-201.

(60) Bangor A, Kortum P, Miller J. An emperical evaluation of the system usability scale. International Journal of Human-Computer Interaction 2008;24:574-594.

(61) Brierley G, Brabyn S, Torgerson D, Watson J. Bias in recruitment to cluster randomized trials: a review of recent publications. J Eval Clin Pract 2012 Aug;18(4):878-86.

(62) Boter H, van Delden JJ, de Haan RJ, Rinkel GJ. Modified informed consent procedure: consent to postponed information. BMJ 2003 Aug;2(327(7409)):284-5.

(63) van der Graaf R, Koffijberg H, Grobbee DE, de Hoop E, Moons KG, van Thiel GJ, et al. The ethics of cluster-randomized trials requires further evaluation: a refinement of the Ottawa Statement. J Clin Epidemiol 2015 Sep;68(9):1108-14.

15. APPENDIX 1

104 ICPC codes refer to MUPS related diagnoses

Abdomen

D01 Abdominal pain/ cramps general

D02 Abdominal pain epigastric

D04 Rectal/ anal pain

D06 Abdominal pain localized other

D08 Flatulence/ gas/ belching

D09 Nausea

D11 Diarrhoea

D12 Constipation

D18 Change faeces/ bowel movements

D93 Irritable bowel syndrome

T03 Loss of appetite

T08 Weight loss

Fatigue

A04 Weakness/tiredness general

.01 Chronic fatigue syndrome

Musculoskeletal

L01 Neck symptom/ complaint

L02 Back symptom/ complaint

L03 Low back symptom/ complaint

L05 Flank symptom/ complaint

L06 Axilla symptom/ complaint

L07 Jaw symptom/ complaint

L08 Shoulder symptom/ complaint

L09 Arm symptom/ complaint

L10 Elbow symptom/ complaint

L11 Wrist symptom/ complaint

L12 Hand/ finger symptom/ complaint

L13 Hip symptom/ complaint

L14 Leg/ thigh symptom/ complaint

L15 Knee symptom/ complaint

L16 Ankle symptom/ complaint

L17 Foot/ toe symptom/ complaint

L18 Muscle pain

.01 Fibromyalgia

L79 Sprain/ strain of joint NOS

.01 Whiplash trauma cervical spine

Cardiology-Respiratory

K01 Heart pain

K02 Pressure/ tightness of heart

K03 Cardiovascular pain NOS

K04 Palpitations/ awareness of heart

K05 Irregular heartbeat other

L04 Chest symptom/ complaint

(Pseudo-)Neurology and ENT

A01 Pain general/ multiple sites

F13 Eye sensation abnormal

H02 Hearing complaint

H03 Tinnitus, ringing/buzzing ear

N01 Headache

N02 Tension headache

N03 Pain face

N05 Tingling fingers/feet/toes

N17 Vertigo/dizziness

.01 Sensation of unsteadiness

.02 Lightheadedness

Other

S01 Pruritis

R98 Hyperventilation syndrome

Psychiatry

A26 Fear of cancer NOS

A27 Fear of other disease NOS

B25 Fear of aids/ HIV

B26 Fear cancer blood/ lymph

B27 Fear blood/ lymph disease other

D26 Fear of cancer of digestive system

D27 Fear of digestive disease other

F27 Fear of eye disease

H27 Fear of ear disease

K24 Fear of heart disease

K25 Fear of hypertension

K27 Fear cardiovascular disease other

L26 Fear of cancer musculoskeletal

L27 Fear musculoskeletal disease other

N26 Fear cancer neurological system

N27 Fear of neurological disease other

P01 Feeling anxious/nervous/tense

P06 Sleep disturbance

P75 Somatization disorder

R26 Fear of cancer respiratory system

R27 Fear of respiratory disease other

S26 Fear of cancer of skin

S27 Fear of skin disease other

T26 Fear of cancer of endocrine system

T27 Fear endocrine/metabolic dis other

U26 Fear of cancer of urinary system

U27 Fear of urinary disease other

X23 Fear sexually transmitted disease (f)

X24 Fear of sexual dysfunction female

X25 Fear of genital cancer female

X26 Fear of breast cancer female

Y24 Fear of sexual dysfunction male

Y25 Fear sexually transmitted dis. Male

Y26 Fear of genital cancer male

Y27 Fear of genital disease male other

Z29.01 Burnout / stress

Urological/ Genital complaints

U02 Urinary frequency/urgency

U05 Urination problems other

X01 Genital pain female

X02 Menstrual pain

X03 Intermenstrual pain

X04 Painful intercourse female

X09 Premenstrual symptom/complaint

X11 Menopausal symptom/complaint

X15 Vaginal symptom/complaint other

X16 Vulval symptom/complaint

X17 Pelvis symptom/complaint female

Y01 Pain in penis

Y02 Pain in testis/scrotum

Y04 Penis symptom/complaint other

Y08 Sexual function symptom/ complaint (m)

16. APPENDIX 2

Intake	Physical therapist	Anamnesis and physical examination<br>Providing information about the online part of the PARASOL intervention
	Mental health nurse	Anamnesis according to the SCEGS
Week 1	Physical therapist	Education about central sensitisation<br>Providing information about the 3-day baseline self-test
	Mental health nurse	Education about the consequence model
	Patient	Online module 1:<br>- what is central sensitisation?<br>- what is the consequence model?<br>Performance of a 3-day baseline test
Week 2	Patient	Online module 2:<br>- Graded activity<br>- Behavioral change
Week 3	Physical therapist	Evaluation of education week 1<br>Education about graded activity<br>Evaluation results from the 3-day self-test<br>Determining short term goal<br>Discussing the gradual increase of the selected activity
	Mental health nurse	Patient specific inventory on the consequence model<br>Education about coping strategies
	Patient	Online module 3:<br>- Starting gradually increase selected activity
Week 4	Patient	Online module 4:<br>- Stress
Week 5	Patient	Online module 5:<br>- Coping with physical complaints
Week 6	Physical therapist	Evaluation of graded activity<br>Evaluation of online modules<br>Coaching on lifestyle
	Mental health nurse	Evaluation of the consequence model<br>Evaluation of the coping strategies
	Patient	No online module

Week 7	Patient	Online module 7:<br>- Relaxation
Week 8	Patient	Online module 8:<br>- Lifestyle advises
Week 9	Patient	Online module 9:<br>- Creating an exercise plan
Week 10	Patient	Online module 10:<br>- Performing the exercise plan
Week 11	Patient	Online module 11:<br>- How to stay active and avoiding a relapse
Week 12	Physical therapist	Discussing long-term goals<br>Support to maintain a physically active lifestyle
